# Fine Tuning the Glass Transition Temperature and Crystallinity by Varying the Thiophene-Quinoxaline Copolymer Composition

**DOI:** 10.3390/ma17246031

**Published:** 2024-12-10

**Authors:** Xun Pan, Mats R. Andersson

**Affiliations:** 1 Flinders Institute for NanoScale Science and Technology, College of Science and Engineering, Flinders University, Sturt Road, Bedford Park, Adelaide, SA 5042, Australia; caroline.pan@flinders.edu.au; 2 ARC Training Centre for Biofilm Research and Innovation, Flinders University, Bedford Park, Adelaide, SA 5042, Australia

**Keywords:** conjugated polymers, side-chain modification, copolymer composition, organic photovoltaics, glass transition temperatures, crystallinity

## Abstract

In recent years, the design and synthesis of high-performing conjugated materials for the application in organic photovoltaics (OPVs) have achieved lab-scale devices with high power conversion efficiency. However, most of the high-performing materials are still synthesised using complex multistep procedures, resulting in high cost. For the upscaling of OPVs, it is also important to focus on conjugated polymers that can be made via fewer simple synthetic steps. Therefore, an easily synthesised amorphous thiophene−quinoxaline donor polymer, TQ1, has attracted our attention. An analogue, TQ-EH that has the same polymer backbone as TQ1 but with short branched side-chains, was previously reported as a donor polymer with increased crystallinity. We have synthesised copolymers with varied ratios between octyloxy and branched (2-ethylhexyl)oxy-substituted quinoxaline units having the same polymer backbone, with the aim to control the aggregation/crystallisation behaviour of the resulting copolymers. The optical properties, glass transition temperatures and degree of crystallinity of the new copolymers were systematically examined in relation to their copolymer composition, revealing that the composition can be used to fine-tune these properties of conjugated polymers. In addition, multiple sub-*T*_g_ transitions were found from some of the polymers, which are not commonly or clearly seen in other conjugated polymers. The new copolymers were tested in photovoltaic devices with a fullerene derivative as the acceptor, achieving slightly higher performances compared to the homopolymers. This work demonstrates that side-chain modification by copolymerisation can fine-tune the properties of conjugated polymers without requiring complex organic synthesis, thereby expanding the number of easily synthesised polymers for future upscaling of OPVs.

## 1. Introduction

The development of conjugated polymers has experienced a breakthrough since the introduction of the donor−acceptor (D−A) approach, which was achieved by combining electron-rich (donor) and electron-deficient (acceptor) moieties [1]. The D−A architecture allows for fine-tuning the energy levels of polymers, which is beneficial for the absorption of light and charge transport, both important properties of materials that can be used in organic photovoltaics (OPVs). 

In order to successfully apply a conjugated polymer as an electron donor material in an active layer in an OPV device, the donor polymer needs to have matched energy levels with the acceptor polymer/molecule [2,3], solution processability [4,5], intermixed with acceptor at the macroscale and formation of phase separation at the nanoscale [6,7,8], all of which are crucial for the charge separation and transport in the device [9,10,11]. Besides the D−A approach, another strategy to achieve designed properties of a conjugated polymer is side-chain modification.

Side-chain modification plays a crucial role in the design and optimization of organic materials, especially in polymers applied in OPVs [12,13,14,15,16,17]. One of the primary roles of side-chains is to enhance the solubility of the polymer or small molecules in common solvents. This is crucial for solution processing techniques such as spin-coating or roll-to-roll printing. Side-chain modification can also influence the crystallinity of conjugated polymers, attributed to the effect of side-chains on the packing of polymeric backbones.

Since the development of non-fullerene acceptors (NFAs), the power conversion efficiencies (PCEs) of OPVs have kept increasing over the past 5 years, reaching over 20% in the laboratorial scale cell [18,19]. However, the cost of NFAs is very high, which contradicts to the concept of low-cost that OPVs are claimed to offer [20]. In addition, before the long-term stability of NFAs can be achieved, the application of NFAs in large-scale printed OPVs is unapproachable. Under this circumstance, reviewing fullerene-based solar cells could bring out potential candidate donor polymers that are suitable for the upscaled fabrication of OPVs in the near future.

TQ1, an amorphous polymer composed of thiophene and quinoxaline moieties, is a good example of a donor polymer developed utilising the D−A approach [21]. This polymer has been reported to offer a 6.5% PCE when paired with a fullerene derivative, PC_71_BM, with an additive and poly(4-vinylpyridine)-modified ZnO interface, and has also been successfully applied in electrochromic devices [22,23]. The biggest advantage of this polymer is that it can be easily synthesised and upscaled with low cost, which make it highly relevant for upscaled synthesis for the future upscaling of OPVs [24]. Moreover, TQ1-PCBM and pure TQ1 can be successfully converted into aqueous dispersed nanoparticles for more environmentally friendly preparation of OPVs [25,26] and electrochromic devices [27], respectively.

**Figure 1:**
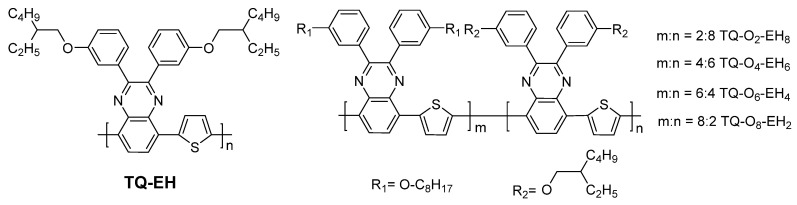
Chemical structures of TQ-EH and copolymers with different loadings of octyloxy and (2-ethylhexyl)oxy side-chains.

However, TQ1 has a relatively low glass transition temperature (*T*_g_) that is non-ideal for the long-term stability of solar cells [28,29]. TQ-EH, another thiophene−quinoxaline (TQ) polymer with significantly higher *T*_g_ and higher crystallinity, but with lower solubility and performance in OPVs, has previously been described [30]. In this work, we have combined the use of linear side-chains and short branched side-chains in copolymers with various ratios with the aim to increase the *T*_g_ and the crystallinity/aggregation compared to the amorphous polymer with only linear side-chains (TQ1) and increased solubility compared to TQ-EH. The designed copolymers (Figure 1) were successfully synthesized and characterized to compare their optical, thermal and mechanical properties, along with their degree of crystallinity. Finally, the new polymers were tested in OPVs to evaluate their photovoltaic performances, revealing a slight increase in performance compared to the homopolymers. The morphology of the active layers was examined to correlate with the observed differences in performance.

## 2. Experimental

### 2.1. Materials and Synthesis

All chemical reactions were carried out under nitrogen protection. Chemicals were purchased from Sigma-Aldrich (Darmstadt, Germany) and ChemSupply Australia (Adelaide, Australia) and were used without further purification. 5,8-dibromo-2,3-bis(3-(octyloxy)phenyl)quinoxaline and 2,5-bis(trimethylstannyl)thiophene were purchased from Solarmer Material Inc. (Beijing, China) and were purified by recrystallisation from iso-propanol and methanol, respectively. All solvents were used as obtained from the suppliers, except for toluene, which was dried and distilled prior to the polymerisation reactions. The monomer 5,8-dibromo-2,3-bis(3-((2-ethylhexyl)oxy)phenyl)quinoxaline was synthesised through the Williamson etherification between compounds 3,3′-(5,8-dibromoquinoxaline-2,3-diyl)diphenol and 2-ethylhexyl bromide; the detailed synthesis can be found in the supporting information. The polymers were synthesised via the Stille coupling polymerisation using the monomers 5,8-dibromo-2,3-bis(3-(octyloxy)phenyl)quinoxaline, 5,8-dibromo-2,3-bis(3-((2-ethylhexyl)oxy)phenyl)quinoxaline and 2,5-bis(trimethylstannyl)thiophene with feed ratios detailed in the discussion. The detailed conditions can be found in the supporting information.

### 2.2. Material Characterisation

The ^1^H-NMR spectra were measured using a Bruker 600 MHz NMR spectrometer (supplied by Bruker, Billerica, MA, USA). The recorded ^1^H NMR were referred to residual solvent peaks.

Gel-permeation chromatography (GPC) measurements were conducted using an Agilent 1260 Infinity II High Temperature GPC System (supplied by Agilent, Santa Clara, USA). 1,2,4-trichlorobenzene was used as an eluent under the operating temperature at 150 °C. The molecular weights were calculated by relative calibration with polystyrene standards.

Cyclic voltammetry (CV) measurements were performed on an AUTOLAB PGSTAT potentiostat (Metrohm AG, Herisau, Switzerland) using a three-electrode setup with platinum wires, both for a working electrode, a counter electrode and a Ag/Ag^+^ reference electrode. Solutions of tetrabutylammonium hexafluorophosphate (Bu_4_NPF_6_) in acetonitrile (0.1 M) were used as supporting electrolytes. Energy levels of the materials were calculated by setting the peak potential of Fc/Fc^+^ vs. the normal-hydrogen electrode (NHE) to 0.630 V and the NHE vs. the vacuum level to 4.5 V [31]. The HOMO and LUMO energy levels were calculated according to the formula HOMO = −($E_{ox}^{onset}$ + 5.13) eV and LUMO = −($E_{red}^{onset}$ + 5.13) eV, where $E_{ox}^{onset}$ and $E_{red}^{onset}$ are determined from oxidation and reduction onsets in cyclic voltammograms, respectively.

Dynamic mechanical thermal analysis (DMTA) measurements were carried out on a TA Q800 DMA (supplied by TA instruments, New Castle, DE, USA) in a strain-controlled mode at a frequency of 1 Hz and an amplitude of 5 µm. The sample preparation and the details of DMTA measurements were according to previous reported procedures [28,31].

Differential scanning calorimetry (DSC) measurements were performed on a TA Instruments Discovery DSC 250 (New Castle, DE, USA) under a 50 mL/min nitrogen flow. The samples were firstly heated from r.t. to 350 °C, then cooled to −80 °C to remove thermal history, followed by the second heating to 380 °C, and then cooled to r.t. The heating and cooling rates were both 10 °C/min. The DSC curves presented were taken from the second heating and cooling scans. The melting enthalpy values were calculated from the second heating scan and used to compare the degree of the crystallinity of the polymers.

Atomic force microscopy (AFM) (supplied by Bruker, Billerica, MA, USA) was performed in tapping mode using silicon tips to probe the morphology of active layers. The films were prepared by spin-coating from the same polymer−PC_71_BM solutions used for device fabrication on cleaned ITO-glass substrates.

### 2.3. Organic Photovoltaics Fabrication

Inverted solar cells were fabricated with an ITO/ZnO/BHJ/MoO_3_/Ag structure. Patterned ITO-coated glass substrates (10 Ω/sq; purchased from Xin Yan Technology Ltd., Hong Kong, China) were cleaned using the procedure reported [22,32]. The ZnO layer was prepared via the deposition of zinc oxide sol-gel [33] on the ITO substrate, followed by annealing at 280 °C for 10 min in air to yield a 25 nm-thick film. The BHJ layers were spin-coated from 25 mg/mL polymer:PC_71_BM (1:2.5 weight ratio) *o*-DCB solutions in a nitrogen-filled glove-box at 500 rpm for 1 min followed by 2000 rpm for 30 s. After the deposition of BHJ layers, a 12 nm MoO_3_ layer was thermally evaporated under 1.5 ×10^−6^ torr using a Covap system supplied by Angstrom Engineering. Finally, an 80 nm Ag electrode was thermally evaporated through a shadow mask, which defined the active area to be 0.1 cm^2^.

### 2.4. Organic Photovoltaics Characterisation

An Oriel solar simulator (supplied by NewSpec Pty Ltd, Adelaide, Australia) fitted with a 150 W xenon lamp (Newport) was used to provide simulated solar light, giving an irradiation of 100 mW/cm^2^ at AM1.5. The walking distance between OPVs and the solar light was calibrated using a silicon reference cell (supplied by NewSpec Pty Ltd, Adelaide, Australia) with NIST traceable certification. The photocurrent density–voltage (*J−V*) characteristics of OPV devices were measured in air through a Keithley 2400 SourceMeter (supplied by Tektronix, Beaverton, OR, USA).

## 3. Results and Discussions

Conjugated polymers that are designed using the donor:acceptor (D:A) approach have been widely reported, and TQ1 that contains thiophene as the donor block and quinoxaline as the acceptor moiety is one of the examples of D:A polymers that is easy to synthesis [34]. 

In order to understand the influence of side-chains attached to the quinoxaline unit of thiophene−quinoxaline (TQ), several TQ-based polymers were synthesised, and their thermal properties were studied, especially the crystallinity and glass transition. Quinoxaline monomers that were employed in this study were 5,8-dibromo-2,3-bis(3-(octyloxy)phenyl)quinoxaline (Q-O) and 5,8-dibromo-2,3-bis(3-((2-ethylhexyl)oxy)phenyl)quinoxaline (Q-EH), with the later one synthesised following the reported procedures [35], with detailed synthesis in the supporting information, Scheme S1 and NMR spectra shown in Figure S1. TQ-EH was synthesised by the Stille coupling polymerisation using the monomers Q-EH and 2,5-bis(trimethylstannyl)thiophene. Copolymers with varied contents of the octyloxy and branched (2-ethylhexyl)oxy (EH) side-chains were polymerised using three monomers, Q-O, Q-EH and 2,5-bis(trimethylstannyl)thiophene with different molar ratios. The details of the polymerisations can be found in the supporting information, Scheme S2. The ratios between octyloxy and EH side-chains discussed in this study were based on the molar feed ratios between Q-O and Q-EH monomers in the polymerisation, which are listed in Table S1 in the supporting information.

The actual ratios of Q-O to Q-EH units in the copolymers were determined using the integrals of protons (-O-CH2-) from the 1H NMR spectra (see the supporting information: Figures S2–S4). These ratios aligned closely with the molar feed ratios of the quinoxaline monomers.

Although the octyloxy and (2-ethylhexyl)oxy side-chains contains the same number of carbon atoms, the TQ-EH polymer that was only substituted with branched side-chains could only be dissolved in hot chlorobenzene (CB) or ortho-dichlorobenzene (*o*-DCB), indicating low solubility in other solvents normally used in preparing active layers of OPVs.

During the purification of TQ-O2-EH8 and TQ-O4-EH6, we have found that both polymers had a relatively small amount of low molecular weight fractions (detailed in the supporting information) that can be extracted by CHCl_3_, while the high molecular weight fractions were extracted by *o*-DCB. With the inclusion of more linear octyloxy side-chain in the copolymers, TQ-O6-EH4 and TQ-O8-EH2 both showed very good solubility in CHCl_3_. The molecular weights of the polymers were measured using high-temperature GPC with 1,2,4-trichlorobenzene as an eluent, and the values are listed in Table 1 (see the supporting information: Figures S5–S10 for GPC traces). The polymers presented in this work all showed similar molecular weights; hence, the different solubility of these polymers resulted from the different ratios between branched and linear side-chains, and a higher proportion of linear side-chains provided better solubility of polymers in commonly used solvents.

### 3.1. Optical Properties of Copolymers

**Figure 2:**
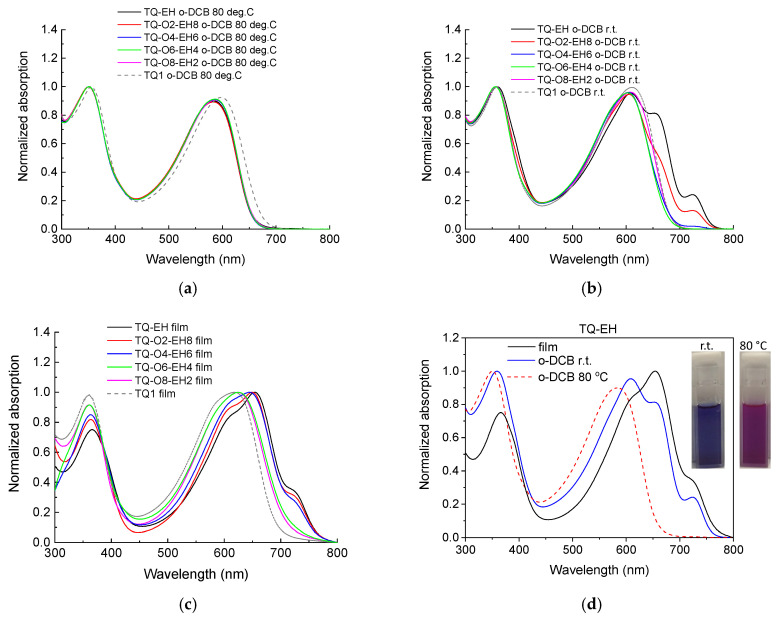
UV−vis spectra of polymers TQ-EH, TQ-O2-EH8, TQ-O4-EH6, TQ-O6-EH4, TQ-O8-EH2 and TQ1 in the 80 °C *o*-DCB solutions (**a**), *o*-DCB solutions at r.t. (**b**) and solid films (**c**). (**d**) The UV−vis spectra of TQ-EH in different conditions with the inserted photo showing the colours of TQ-EH *o*-DCB solutions at different temperatures.

Side-chain modification has been known to affect the packing and aggregation of the polymers, which can be reflected and examined from the materials’ optical properties. Hence, we recorded the UV−vis spectra of the polymers at 80 °C and at room temperature (r.t.) in *o*-DCB, as well as the polymers in the solid-state films, and the results are shown in Figure 2. TQ1 *o*-DCB solutions and film were also measured and presented here for a direct comparison. All polymers shown this work showed a very similar absorption at 80 °C in *o*-DCB solutions with two broad peaks, while at r.t. in *o*-DCB, TQ-EH and TQ-O2-EH8 they exhibited extra peaks in a high wavelength region and red-shifted spectra, indicating the existence of aggregates for both polymers at low concentrations. In the solid states, TQ1, TQ-O8-EH2 and TQ-O6-EH4 showed slightly red-shifted spectra compared to the spectra of their solutions. In the case of polymers with a higher proportion of branched side-chains, the UV−vis spectra from their solutions to the solid films experienced pronounced redshifts, suggesting that with an increased amount of (2-ethylhexyl)oxy side-chains, polymers have a higher tendency to form aggregates in the solid state. The comparisons of UV−vis spectra of TQ-EH and the colours of its solutions at different temperatures are presented in Figure 2d, and a significant red-shifted absorption onset of 113 nm from its hot *o*-DCB solution to the film was revealed, agreeing with the previous finding that TQ-EH has a high tendency to form aggregates/crystals [30]. The comparison of other polymers in different conditions can be found in the supporting information (Figure S11), and the optical characteristics are summarized and listed in Table 2.

### 3.2. Electrochemical Properties of Copolymers

One key property of conjugated polymers for the application of organic electronics is the energy levels. As TQ1 and TQ-EH have been shown to function as donor polymers in fullerene-based OPVs, the potential application of their derivative copolymers is a donor component in OPVs. Before examining the photovoltaic performances of the copolymers, we have carried out electrochemistry measurements to study the energy levels. The cyclic voltammograms can be found in the supporting information (Figure S12), and the onset potential values as well as HOMO and LUMO energy levels are listed in Table 3. The five polymers that contained different proportions of (2-ethylhexyl)oxy side-chains showed near identical energy levels, with less than 0.1 eV differences in their HOMO and LUMO levels. The bandgap values estimated from CV agreed well with the optical bandgap of the polymers shown in Table 2. The electrochemistry results suggest that the side-chain modification employed in this study had a negligible effect on the energy levels of this series of thiophene−quinoxaline polymers, which matched well with the energy levels of the commercially available fullerene derivative, PC_71_BM [36]. 

### 3.3. Thermal Transitions and the Degree of Crystallinity

**Figure 3:**
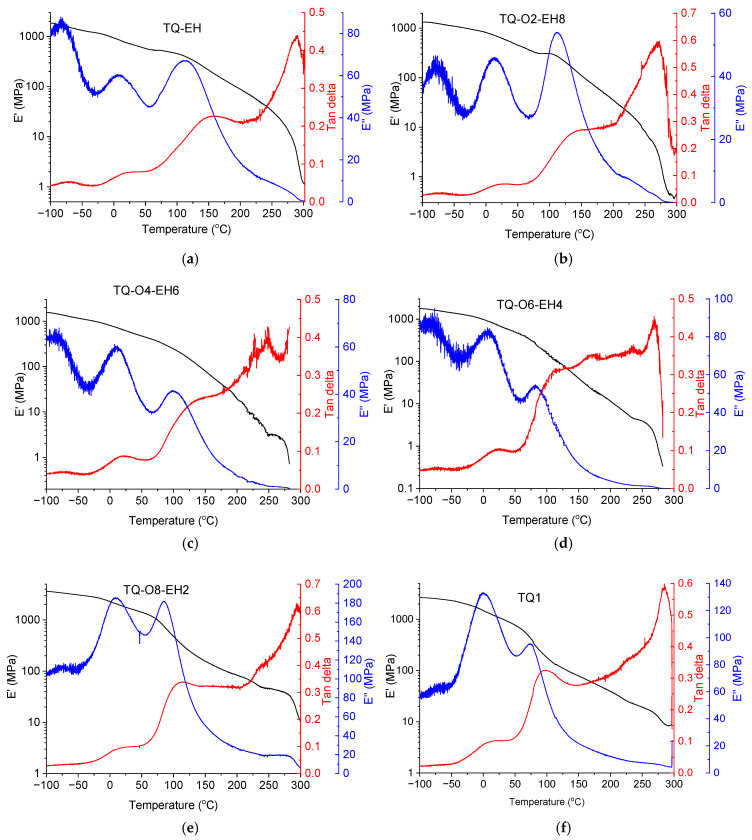
DMTA plots of polymers: (**a**) TQ-EH; (**b**) TQ-O2-EH8; (**c**) TQ-O4-EH6; (**d**) TQ-O6-EH4; (**e**) TQ-O8-EH2; (**f**) TQ1. The DMTA samples of polymers TQ-O4-EH6 and TQ-O6-EH4 broke at ~280 °C.

To understand the thermal properties of this series of copolymers, especially glass transition temperatures (*T*_g_), DMTA measurements were carried out, and the results are presented in Figure 3. Including the reported TQ1 and TQ-EH, all six polymers measured showed clear thermal transitions between 50 and 200 °C, revealed from clear peaks in loss moduli (E”) at high temperatures accompanied by big drops in storage moduli (E’) starting from approximate 100 °C. The peak temperatures from the tan delta curves were used to assign the thermal transition temperatures that are listed in Table 4. *T*_α_, *T*_β_ and *T*_γ_ present the transition temperatures from high to low states for simplifying the discussion here, where *T*_α_ is highly likely to be the *T*_g_ according to our previous findings [31,37].

The DMTA result of TQ1 showed clear thermal transitions at 97 and 12 °C (Figure 3f), which agreed with the previously reported *T*_g_ of 95 °C and the sub-*T*_g_ of 10 °C for TQ1, respectively, when probed using the same technique [28]. With an increase of the EH side-chain content from 20% to 100% in the polymers, the *T*_g_ values increased from 109 °C for TQ-O8-EH2 to 153 °C for TQ-EH, while *T*_β_ only experienced a slight increase (Table 4). The similar *T*_β_ temperatures suggest that similar thermal energy is needed for the thermal relaxation of the side-chains that contains either octyloxy or (2-ethylhexyl)oxy groups. The higher *T*_g_ values found in the polymers with higher amounts of EH side-chains suggests that the inclusion of more EH groups enhances the rigidity of polymer backbone, which may relate to the higher tendency to form aggregates as revealed by UV−vis results. It should be noted that the samples prepared from TQ-O4-EH6 (Figure 3c) and TQ-O6-EH4 (Figure 3d) could not be measured up to 300 °C and broke at approximately 280 °C due to a very low mechanical strength, which could be a result from the melting of polymers and will be discussed later in this work. 

**Figure 4:**
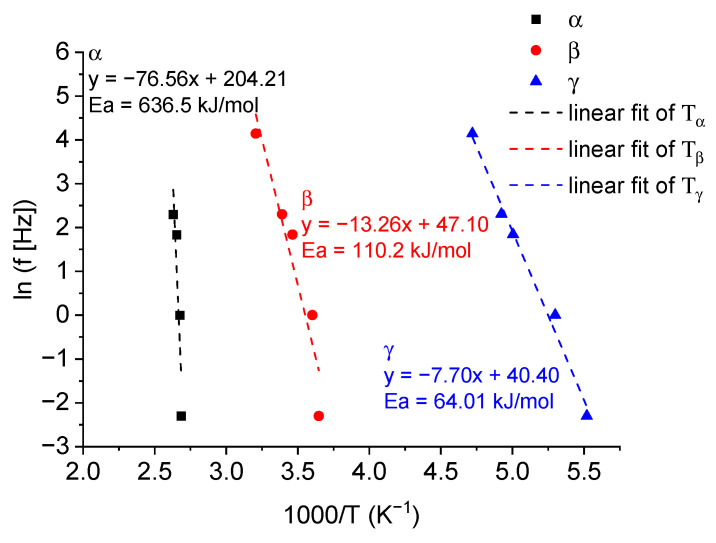
Arrhenius plot of TQ-O2-EH8 showing the linear fits and resulting activation energies for the thermal transitions. The dashed lines represent the linear fit of the scatter plot.

Besides the findings that the copolymers showed different values of *T*_g_, we also found one extra thermal transition at a very low temperature of approximately −70 °C in some of the polymers, which are not commonly seen in other conjugated polymers [38,39]. To be more specific, three thermal transitions can be clearly seen from the E” curves of TQ-EH, TQ-O2-EH8, TQ-O4-EH6 and TQ-O6-EH4, and the high temperature thermal transitions between 100 and 160 °C are highly likely to be the glass transition of these polymers, based on the reported *T*_g_ of TQ1. To further confirm this as well as that the low temperature peaks in the E” are associated with the sub-*T*_g_ thermal relaxation, the frequency of applied strain was varied in the DMTA instrument for a TQ-O2-EH8 sample, and it was measured at 0.1, 1, 6.3 and 10 Hz separately. The DMTA plots with varied frequencies are shown in the supporting information (Figures S13–S16). Peaking fitting of E” curves was performed using a Gaussian model, and the fitted peak temperatures were used to estimate the activation energy (Ea) using the Arrhenius kinetics equation [38,40] $\ln\left(f\right)=-\frac{E_{a}}{R}\left(\frac{1}{T}\right)+lnA$, where f is the frequency, R is the gas constant, T is the transition temperature in Kelvin and A is the pre-exponential factor. Figure 4 shows the Arrhenius plot of TQ-O2-EH8, where *T*_α_, *T*_β_ and *T*_γ_ are the transition temperatures from high to low states, respectively. Linear fitting was carried out, and the fitting results are inserted in Figure 4. The Ea of α transition was 6 times higher than the β transition and one magnitude higher than the γ transition, confirming *T_α_* was *T*_g_ [31]. The origin of β and γ transitions could be the localised molecular movements, involving side-chains or smaller segments of the polymer, which required lower thermal energy than the glass transition.

Since TQ-EH was reported to have a crystalline phase [30], it is expected that the copolymers based on the same polymeric backbone may also have a certain degree of crystallinity. Therefore, DSC measurements were performed to compare the degree of crystallinity of the polymers. The DSC thermograms of the polymers can be found in the supporting information (Figures S17–S22). The melting enthalpy (Δ*H*_m_) values were obtained by integrating the melting peaks between 230 to 310 °C on the second heating thermograms and are listed in Table 4; the melting peak temperatures can be found in the supporting information (Table S2). These melting temperatures were confirmed by the large decrease in E’ in the DMTA measurements of the polymers. In addition, all polymers were found to have a liquid-crystalline-to-isotropic transition at approximately 350 °C, agreeing with previous findings [30]. The corresponding enthalpy (Δ*H*_LC)_ and peak temperatures (*T*_LC_) are listed in the supporting information (Table S2).

The polymer that was substituted with 100% EH side-chains, TQ-EH, had a Δ*H*_m_ of 7.84 J/g. The Δ*H*_m_ values (Table 4) were found to decrease with a lower EH side-chain content, following a nearly linear trend. Since the degree of crystallinity is proportional to the amount of energy absorbed during melting, a higher melting enthalpy indicates a greater proportion of crystalline regions, leading to a higher degree of crystallinity. Therefore, the DSC results clearly revealed that TQ polymers with a lower EH side-chain content studied in this work had a lower degree of crystallinity. TQ1, which contained only linear octyloxy side-chains, only showed a liquid-crystalline-to-isotropic transition at 353 °C. It is worth noting that TQ-O4-EH6 and TQ-O6-EH4 both had two separated melting peaks, suggesting that two different crystallites existed in these polymers. The lower temperature melting of these two polymers peaked at 247 and 230 °C, respectively, explaining that the breaking of their DMTA samples at ~280 °C was due to the melting of part of the polymers, which greatly reduced their mechanical strength. Furthermore, no clear *T*_g_ or sub-*T*_g_ was found from the DSC thermograms, confirming that DMTA was much more sensitive for the study of *T*_g_ and sub-*T*_g_ transitions in conjugated polymers.

The DMTA and DSC results demonstrate that copolymer composition can fine tune the thermal transition temperatures and the degree of crystallinity of conjugated polymers.

### 3.4. Device Performance

The electrochemistry measurements have revealed the energy levels of these polymers to be suitable as donor materials in OPVs when paired with the fullerene derivative PC_71_BM as the acceptor. Thus, to understand the influence of copolymer composition on the photovoltaic performances of the polymers, inverted OPVs with a structure of ITO/ZnO/active layer/MoO_3_/Ag were fabricated using polymer and PC_71_BM as the active layer. The study of their photovoltaic performance was not to compete with the recorded efficiencies, but rather to develop an understanding of the effect of copolymer composition on the OPVs using these easily synthesised and modified copolymers.

**Figure 5:**
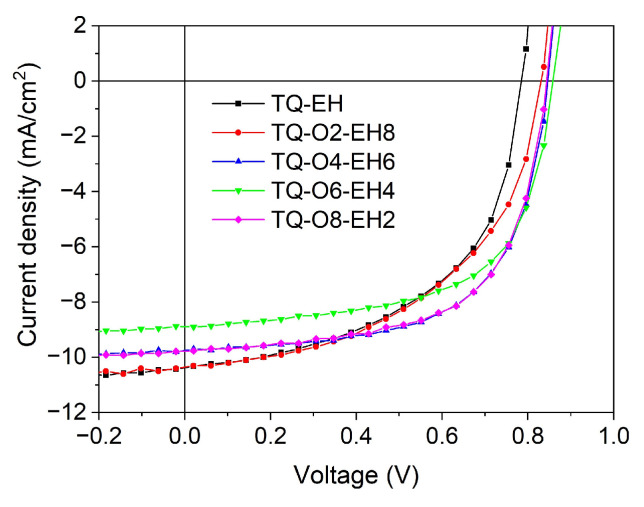
Representative *J−V* curves of polymer:PC_71_BM-based OPVs.

A donor:acceptor ratio of 1:2.5 was used here for all the polymer:PC_71_BM blends, with *o*-DCB as the processing solvent to ensure the complete dissolution of the polymers. Since solvent additives may have different effects on the active layer that contains different polymers, and to exclude the interference arising from the solvent additive, no solvent additive was used during the deposition of the active layers. The device characteristics are listed in Table 5, and the representative J-V curves are shown in Figure 5. The device characteristics of TQ1:PC_71_BM OPVs were taken from previous reported values, which were obtained from the OPVs fabricated using the same configuration without the use of additives [22], for a direct comparison with OPVs made from other TQ polymers.

The devices consisted of a TQ-EH:PC_71_BM active layer showed a *J*_SC_ of 10.37 mA/cm^2^, a FF of 53% and a *V*_OC_ of 0.79 V, resulting in a PCE of 4.34% from the best-performing cell, which was higher than the previously reported PCE of TQ-EH:PC_71_BM devices that were fabricated using a conventional structure [30]. The OPVs made with the TQ-O2-EH8 donor offered similar *J*_SC_, a slightly lower FF of 51% compared to the ones made from TQ-EH, but the higher *V*_OC_ of 0.83 V compensated the loss from FF, and similar PCE was obtained. The TQ-O4-EH6:PC_71_BM devices were found to show the best performances, with the average PCE ± deviation of 4.99 ± 0.09%. The representative external quantum efficiency (EQE) curve from TQ-O4-EH6:PC_71_BM devices can be found in the supporting information (Figure S23). Interestingly, the OPVs with TQ-O8-EH2:PC_71_BM active layers presented nearly identical photovoltaic characteristics as the ones with TQ-O4-EH6:PC_71_BM, indicating the slightly different degrees of crystallinity of donor polymers did not seem to affect the charge extraction ability in the corresponding devices. The OPVs based on the TQ-O6-EH4 donor showed an inferior *J*_SC_ compared to the devices based on other donor polymers, which could be a result of its comparatively lower molecular weight. Although the *J*_SC_ was slightly lower, decent FF and *V*_OC_ were obtained from TQ-O6-EH4:PC_71_BM OPVs, and a PCE of 4.75% was achieved, which was slightly higher than those of the reported TQ1:PC_71_BM devices [22].

Among all the TQ polymers studied in this work, TQ-EH-based OPVs exhibited the lowest *V*_OC_. Since the polymers in this work were found to have very similar HOMO levels and TQ-EH devices exhibited high *J*_SC_, the lower *V*_OC_ of TQ-EH devices compared to the ones made from other copolymers is likely to be a result of a lower charge-transfer (CT) state energy [30]. The FF values gradually increased with the inclusion of more octyloxy side-chains in the donor polymers, while the *J*_SC_ showed an opposite trend, which may be related to the morphology of the polymer:PC_71_BM active layers. 

### 3.5. Morphology Investigation

**Figure 6:**
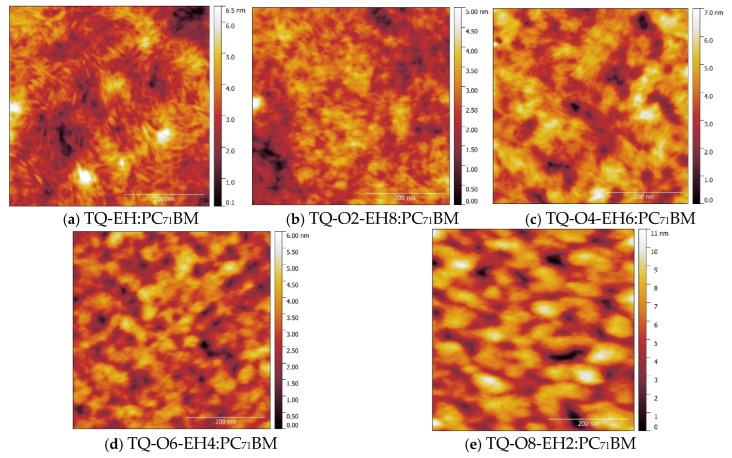
AFM height images (0.5 × 0.5 µm^2^) of polymer:PC_71_BM (1:2.5) films. Scale bars are 200 nm.

The DSC results have shown that polymers with different copolymer compositions have different degrees of crystallinity. The degree of crystallinity of conjugated polymers can influence the morphology of the active layer and result in different charge mobilities and recombination losses [41], thus affecting the photovoltaic performances of OPVs. To investigate the morphology of the polymer−PC_71_BM active layers, AFM measurements were performed on the films that were deposited using the same spin-coating condition as the deposition of the active layer during the device fabrication. The 0.5 × 0.5 µm^2^ AFM height images are shown in Figure 6, and the 5 × 5 µm^2^ height images can be found in the supporting information (Figure S24). The root-mean square roughness (RMS) values calculated from the 5 × 5 µm^2^ height images are listed in Table S3.

The TQ-EH/PC_71_BM film showed fibrillar topographic features (Figure 6a), which could be attributed to the higher crystalline content of TQ-EH. A similar fibrillar feature can also be found in the TQ-O2-EH8:PC_71_BM and TQ-O4-EH6:PC_71_BM films, and the former one seemed to be finer. To further confirm the fibrillar feature was not artefact, the phase images of TQ-EH:PC_71_BM and TQ-O2-EH8:PC_71_BM films were recorded and are shown in the supporting information (Figure S25). The clear fibrillar features observed in the phase images supported the morphology of the fibrillar crystal network. In contrast, the TQ-O6-EH4:PC_71_BM and TQ-O8-EH2:PC_71_BM films were found to be smooth and homogeneous, and no clear crystallites were detected, agreeing with the low degree of crystallinity in TQ-O6-EH4 and TQ-O8-EH2 revealed by DSC results.

The AFM height images at a large scale (Figure S24) suggested the appearance of pin-holes in the TQ-EH:PC_71_BM film, which could negatively affect the balanced charge transport in devices, explaining the low FF of TQ-EH:PC_71_BM-based OPVs. The TQ-O2-EH8:PC_71_BM film was found to have a very low RMS of 0.90 µm, indicating a possible lack of phase separation that is an undesirable morphology for solar cell active layers. On the contrary, TQ-O8-EH2:PC_71_BM film showed an RMS value of 1.94 nm, which was the highest among all the blended films studied here. The high RMS indicates a greater degree of phase separation, as evidenced by the prominent topographic features shown in Figure 6e. This increased phase separation may enhance charge separation in the active layer, leading to a good photovoltaic performance. Furthermore, the AFM results suggest that the good photovoltaic performance of the TQ-O4-EH6:PC_71_BM devices is due to a combination of the fibrillar network and desired phase separation.

## 4. Conclusions

We have modified the side-chains attached to thiophene−quinoxaline polymers with different molar ratios of the octyloxy and (2-ethylhexyl)oxy-substituted quinoxaline moieties. The resulting four new copolymers together with previously reported TQ-EH and TQ1 were systematically studied to understand the influence of copolymer composition on their optical, electrochemical and thermal properties. With the increased proportion of EH-substituted segments in the polymer structure, the polymers showed a higher tendency to form aggregates and had a higher *T*_g_ that is indicative of higher rigidity and enhanced packing. In addition, a higher loading of EH units also resulted in polymers with a higher degree of crystallinity as revealed by DSC results. The OPVs prepared from these copolymers showed slightly higher photovoltaic performances compared to the devices fabricated with homopolymers. The investigation of the active film morphology suggests that the key to achieve good photovoltaic performances is a combination of aggregation/crystallisation and desired phase separation. Overall, our work demonstrates that copolymer composition is a powerful tool that can be used to fine-tune the glass transition temperatures and crystallinity of conjugated polymers.

## Figures and Tables

**Table 1:** Molecular weights of polymers.

Polymer	EH Side-Chain Content	Mn (kg/mol)	Mw (kg/mol)	PDI	Solubility
TQ-EH	100%	28.4	68.9	2.43	80 °C CB, *o*-DCB
TQ-O2-EH8	80%	25.4	69.8	2.75	CB
TQ-O4-EH6	60%	31.2	74.7	2.39	CB
TQ-O6-EH4	40%	21.9	75.7	3.45	CHCl_3_
TQ-O8-EH2	20%	32.2	131.2	4.08	CHCl_3_

**Table 2:** Optical characteristics of polymers with different EH contents.

Polymer	λ_max, sol_ (nm)	λ_onset, sol_ (nm)	λ_max, film_ (nm)	λ_onset, film_ (nm)	Redshift_max_ (nm)	Redshift_onset_ (nm)	$E_{g}^{opt}$ (eV)
TQ-EH	585	655	654	768	69	113	1.61
TQ-O2-EH8	583	655	648	768	65	113	1.61
TQ-O4-EH6	585	657	645	768	60	111	1.61
TQ-O6-EH4	585	660	625	721	40	61	1.72
TQ-O8-EH2	588	655	624	713	36	58	1.74
TQ1	598	675	616	696	18	21	1.78

λ_max, film_—the value in a high wavelength region in the solid state; λ_max, sol_—the value in a high wavelength region in the 80 °C *o*-DCB solution.

**Table 3:** Oxidation and reduction of peak potentials measured from CV as well as the HOMO and LUMO energy levels and bandgaps of polymers.

Polymer	$E_{ox}^{onset}$ (V)	$E_{red}^{onset}$ (V)	HOMO (eV)	LUMO (eV)	*E*g (eV)
TQ-EH	0.30	−1.46	−5.43	−3.67	1.76
TQ-O2-EH8	0.31	−1.43	−5.44	−3.70	1.74
TQ-O4-EH6	0.37	−1.43	−5.50	−3.70	1.80
TQ-O6-EH4	0.37	−1.48	−5.50	−3.65	1.85
TQ-O8-EH2	0.36	−1.47	−5.49	−3.66	1.83

**Table 4:** Thermal transition temperatures of polymers. The *T*_g_, *T*_β_ and *T*_γ_ are peak temperatures of associated peaks. The melting enthalpy (Δ*H*_m_) values were calculated by integrating the melting peaks measured by DSC.

Polymer	EH Side-Chain Content	*T*_g_ (°C)	*T*_β_ (°C)	*T*_γ_ (°C)	Δ*H*_m_ (J/g)
TQ-EH	100%	153	22	−71	7.84
TQ-O2-EH8	80%	137	22	−71	7.39
TQ-O4-EH6	60%	~136	18	~−70	5.77
TQ-O6-EH4	40%	~113	~21	~−70	3.74
TQ-O8-EH2	20%	109	14	-	1.79
TQ1	0%	97	12	-	-

**Table 5:** Characteristics of polymer:PC_71_BM-based OPVs.

Polymer	*J_SC_* (mA/cm^2^)	FF (%)	*V*_OC_ (V)	PCE (%)
TQ-EH	10.37 (9.92 ± 0.27)	53 (52 ± 1)	0.79 (0.79 ± 0.01)	4.34 (4.12 ± 0.11)
TQ-O2-EH8	10.35 (9.81 ± 0.32)	51 (51 ± 0)	0.83 (0.83 ± 0.00)	4.37 (4.12 ± 0.13)
TQ-O4-EH6	9.75 (9.66 ± 0.29)	62 (60 ± 2)	0.86 (0.86 ± 0.00)	5.14 (4.99 ± 0.09)
TQ-O6-EH4	8.89 (8.37 ± 0.28)	62 (62 ± 1)	0.86 (0.86 ± 0.01)	4.75 (4.44 ± 0.16)
TQ-O8-EH2	9.78 (9.02 ± 0.45)	62 (63 ± 1)	0.85 (0.84 ± 0.01)	5.15 (4.79 ± 0.19)
TQ1 *	7.5 ± 0.2	68 ± 2	0.84 ± 0.01	4.7 (4.3 ± 0.3)

* TQ1 device characteristics taken from previously reported results using the same device structure [22].

## Data Availability

The data that support the findings of this study are available from the corresponding author upon reasonable request.

## Supplementary Materials

The following supporting information can be downloaded at: https://www.mdpi.com/article/10.3390/ma17246031/s1, Scheme S1. Synthetic route for the monomer 5,8-dibromo-2,3-bis(3-((2-ethylhexyl)oxy)phenyl)quinoxaline; Table S1. The amounts of monomers used during the polymerisation of copolymers and the resulted polymer amounts.; Scheme S2. Synthetic route for TQ-EH and copolymers.; Figure S1. ^1^H NMR (600 MHz) spectra of 5,8-dibromo-2,3-bis(3-((2-ethylhexyl)oxy)phenyl) quinoxaline.; Figure S2. ^1^H NMR (600 MHz) spectra of TQ1 (green curve), TQ-O8-EH2 (red curve) and TQ-O6-EH4 (blue curve). The quinoxaline units with highlighted (-O-CH_2_-) groups and arrows inserted illustrate the specific proton signals.; Figure S3. ^1^H NMR (600 MHz) spectra of TQ-O6-EH4. The integrals of the (-O-CH_2_-) proton signals reveals that the ratio of Q-O to Q-EH is 6:3.7.; Figure S4. ^1^H NMR (600 MHz) spectra of TQ-O8-EH2. The integrals of the (-O-CH_2_-) proton signals reveals that the ratio of Q-O to Q-EH is 8:2.5.; Figure S5. GPC curve of TQ-EH.; Figure S6. GPC curve of TQ-O2-EH8 *o*-DCB extracts.; Figure S7. GPC curve of TQ-O4-EH6 CHCl_3_ extracts.; Figure S8. GPC curve of TQ-O4-EH6 *o*-DCB extracts.; Figure S9. GPC curve of TQ-O6-EH4.; Figure S10. GPC curve of TQ-O8-EH2.; Figure S11. UV-vis spectra of polymers in different conditions including 80 °C and r.t. *o*-DCB solutions and solid films.; Figure S12. Cyclic voltammograms of copolymers.; Figure S13. DMTA plot TQ-O2-EH8 measured at 0.1 Hz. Peak fitting of E” of TQ-O2-EH8 at 0.1 Hz.; Figure S14. DMTA plot TQ-O2-EH8 measured at 1 Hz. Peak fitting of E” of TQ-O2-EH8 at 1 Hz.; Figure S15. DMTA plot TQ-O2-EH8 measured at 6.3 Hz. Peak fitting of E” of TQ-O2-EH8 at 6.3 Hz.; Figure S16. DMTA plot TQ-O2-EH8 measured at 10 Hz. Peak fitting of E” of TQ-O2-EH8 at 10 Hz.; Figure S17. DSC 2^nd^ heating (red curve) and cooling (green curve) thermograms of TQ-EH.; Figure S18. DSC 2^nd^ heating (red curve) and cooling (green curve) thermograms of TQ-O2-EH8.; Figure S19. DSC 2^nd^ heating (red curve) and cooling (green curve) thermograms of TQ-O4-EH6.; Figure S20. DSC 2^nd^ heating (red curve) and cooling (green curve) thermograms of TQ-O6-EH4.; Figure S21. DSC 2^nd^ heating (red curve) and cooling (green curve) thermograms of TQ-O8-EH2.; Figure S22. DSC 2^nd^ heating (red curve) and cooling (green curve) thermograms of TQ1.; Table S2. Melting transition peak temperature (*T*_m_), the liquid-crystalline to isotropic transition temperature (*T*_LC_) and the corresponding enthalpy (Δ*H*_LC_) obtained from the DSC second heating thermograms.; Figure S23. EQE curve of TQ-O4-EH6:PC_71_BM device.; Figure S24. 5 × 5 µm^2^ AFM height images of polymer:PC_71_BM. Scale bars are 2 µm.; Table S3. root-mean square roughness (RMS) values calculated from the 5 × 5 µm^2^ polymer:PC_71_BM height images.; Figure S25. 1 × 1 µm^2^ AFM height and phase images of TQ-EH:PC_71_BM and TQ-O2-EH8:PC_71_BM films. Scale bars are 400 nm.
