# Supplementary material for: Fine Tuning the Glass Transition Temperature and Crystallinity by Varying the Thiophene-Quinoxaline Copolymer Composition
Source: Materials (Basel). 2024 Dec 10;17(24):6031. doi: 10.3390/ma17246031 (PMC11676980; doi:10.3390/ma17246031)
Supplement: Supplementary file 1 [file materials-17-06031-s001.zip › materials-3303360-supplementary.pdf]

Supporting information

# Fine tuning the glass transition temperature and crystallinity by varying the thiophene-quinoxaline copolymer composition

Xun Pan,[1] Mats R. Andersson*,[1, 2]

[1]Flinders Institute for NanoScale Science and Technology, College of Science and Engineering, Flinders University, Sturt Road, Bedford Park, SA 5042, Australia

[2]ARC Training Centre for Biofilm Research and Innovation, Flinders University, Bedford Park, SA 5042, Australia

## Contents

1. Synthesis details ..... 2
   1.1 Synthesis of monomer ..... 2
   1.2 Synthesis of polymers ..... 2
2. NMR spectra ..... 4
3. GPC traces ..... 6
4. UV-vis spectra of polymers ..... 9
5. Electrochemistry ..... 10
6. DMTA of TQ-O2-EH8 under different frequencies ..... 11
7. DSC thermograms ..... 13
8. EQE of TQ-O4-EH6:$PC_{71}BM$ device ..... 16
9. AFM height and phase images ..... 17
10. References ..... 19

# 1. Synthesis details

## 1.1 Synthesis of monomer

5,8-dibromo-2,3-bis(3-((2-ethylhexyl)oxy)phenyl)quinoxaline

3,3'-(5,8-dibromoquinoxaline-2,3-diyl)diphenol was synthesized according to previous reported procedure.[1] 3,3'-(5,8-dibromoquinoxaline-2,3-diyl)diphenol (0.4285 g, 0.9076 mmol), potassium carbonate (1.254 g, 9.076 mmol), 18-crown-6 (18 mg) were added with 25 mL DMF into a 100 mL round-bottom flask. The mixture was heated up to 80 °C under $N_2$ for 1 hour to yield a dark orange suspension. Then 2-ethylhexyl bromide (1.3189 g, 6.8278 mmol) was dissolved in 5 mL of DMF and the solution was slowly added to the reaction mixture in portions. The reaction mixture was stirred at 80 °C overnight under $N_2$ before adding 50 mL of water to stop the reaction. The resulted mixture was extracted with ethyl acetate, then the organic phase was washed with brine, dried with $MgSO_4$. Then the solvent in filtered solution was removed by rotary evaporation. The crude product was purified through column chromatography with hexane:DCM = 3:1 as the eluent. Yellow viscous liquid was obtained as the target product (0.6 g, 94.9%). $^1$H-NMR (600 MHz, $CDCl_3$) δ 7.91 (s, 2H), 7.18-7.25 (m, 6H), 6.94 (d, 2H), 3.70 (d, 4H), 1.65-1.68 (t, 2H), 1.29-1.41 (m, 16), 0.88-0.92 (m, 12).

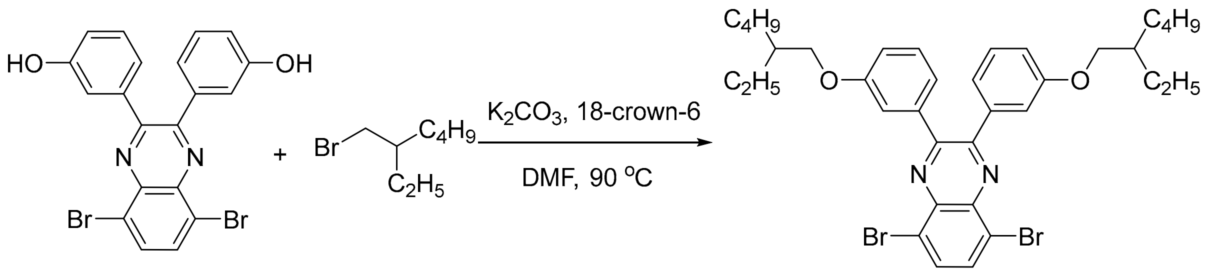


Scheme S1. Synthetic route for the monomer 5,8-dibromo-2,3-bis(3-((2-ethylhexyl)oxy)phenyl)quinoxaline.

## 1.2 Synthesis of polymers

TQ-EH

5,8-dibromo-2,3-bis(3-((2-ethylhexyl)oxy)phenyl)quinoxaline (296.1 mg, 0.425 mmol) and 2,5-bis(trimethylstannyl)thiophene (174.2 mg, 0.425 mmol) were added to a dry 25 mL flask, vacuumed and then protected with Nitrogen before 10 mL of freshly distilled toluene was added to the flask. The solution was stirred at 100 °C under $N_2$ protection for 30 min. Then catalysts, tris(dibenzylideneacetone)dipalladium(0) (7.8 mg) and tri(o-tolyl)phosphine (15.6 mg) were added to the monomer solution. The reaction mixture was stirred at 100 °C for 2 days. Then the solution was precipitated in acetone and filtered to collect solid. The polymer was collected by filtration through an extraction thimble, and Soxhlet-extracted subsequently

with acetone, diethyl ether, ethyl acetate and finally chloroform. It was found only small amounts of polymer was extracted by chloroform and large amounts of polymer solids still remained in the extraction thimble, which was then extracted with *o*-DCB. The chloroform and *o*-DCB extracts were passed through a pad of silica gel, and then precipitated into stirring MeOH (300 mL), respectively. The collected polymers from chloroform and *o*-DCB extracts offered 38.4 mg (14.6%) and 187.5 mg (71.1%), respectively. The chloroform extract should be the low molecular weight fraction and the *o*-DCB extracts were studied in this work.

The copolymers were synthesised and purified following the above-described procedure to make TQ-EH. The detailed amounts of monomers added during the polymerisation are listed in the below table S1. The yield of polymerisation, weights of polymers extracted by chloroform and *o*-DCB are listed below.

**Table S1. The amounts of monomers used during the polymerisation of copolymers and the resulted polymer amounts.**

| | Q-O (mmol) | Q-EH (mmol) | bis(trimethylstannyl) thiophene (mmol) | CHCl3 extracts (mg, yield) | *o*-DCB extracts (mg, yield) |
|---|---|---|---|---|---|
| TQ-O2-EH8 | 0.081 | 0.322 | 0.403 | (23.4, 9.4%)* | 174, 69.5% |
| TQ-O4-EH6 | 0.184 | 0.275 | 0.459 | (163.4, 57.4%)* | 80.3, 28.2% |
| TQ-O6-EH4 | 0.313 | 0.208 | 0.521 | 293.2, 90.6% | - |
| TQ-O8-EH2 | 0.430 | 0.107 | 0.537 | 316.6, 94.8% | - |

*The $CHCl_3$ extracts of TQ-O2-EH8 and TQ-O4-EH6 were not studied in OPVs.

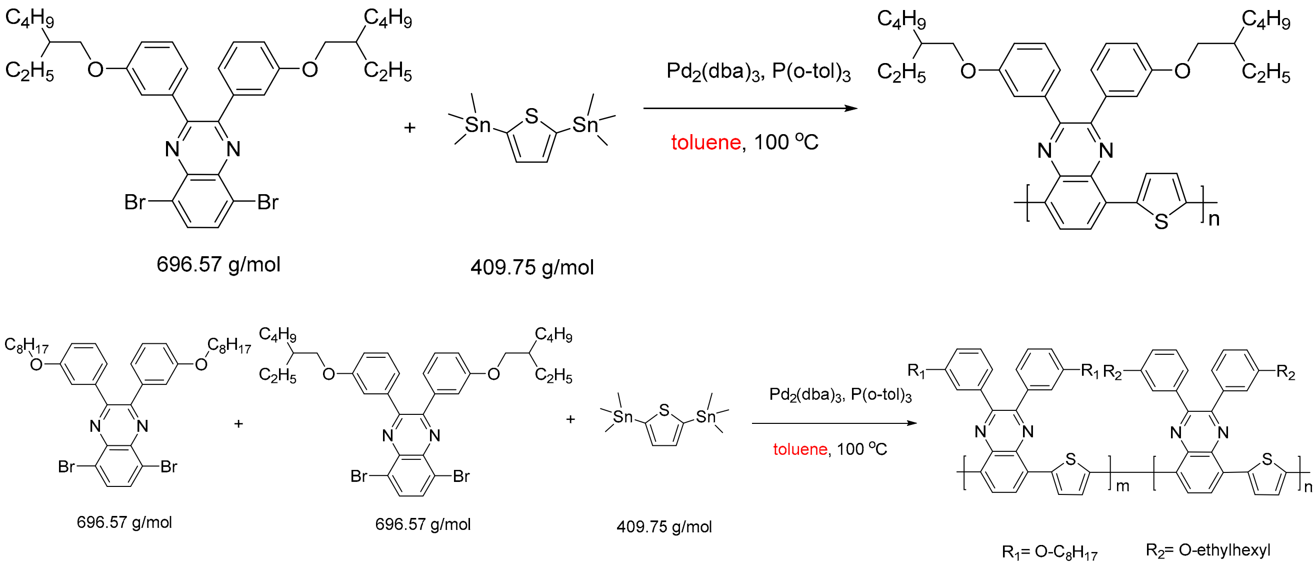


Scheme S2. Synthetic route for TQ-EH and copolymers.

# 2. NMR spectra

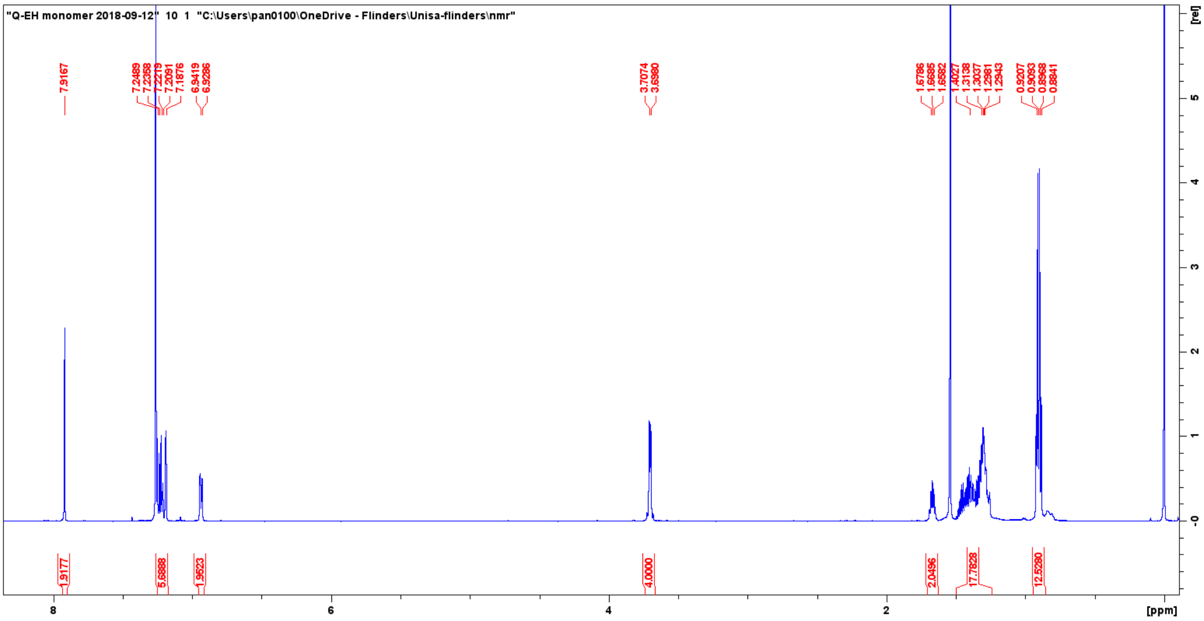


**Figure S1. $^{1}$H NMR (600 MHz) spectra of 5,8-dibromo-2,3-bis(3-((2-ethylhexyl)oxy)phenyl) quinoxaline.**

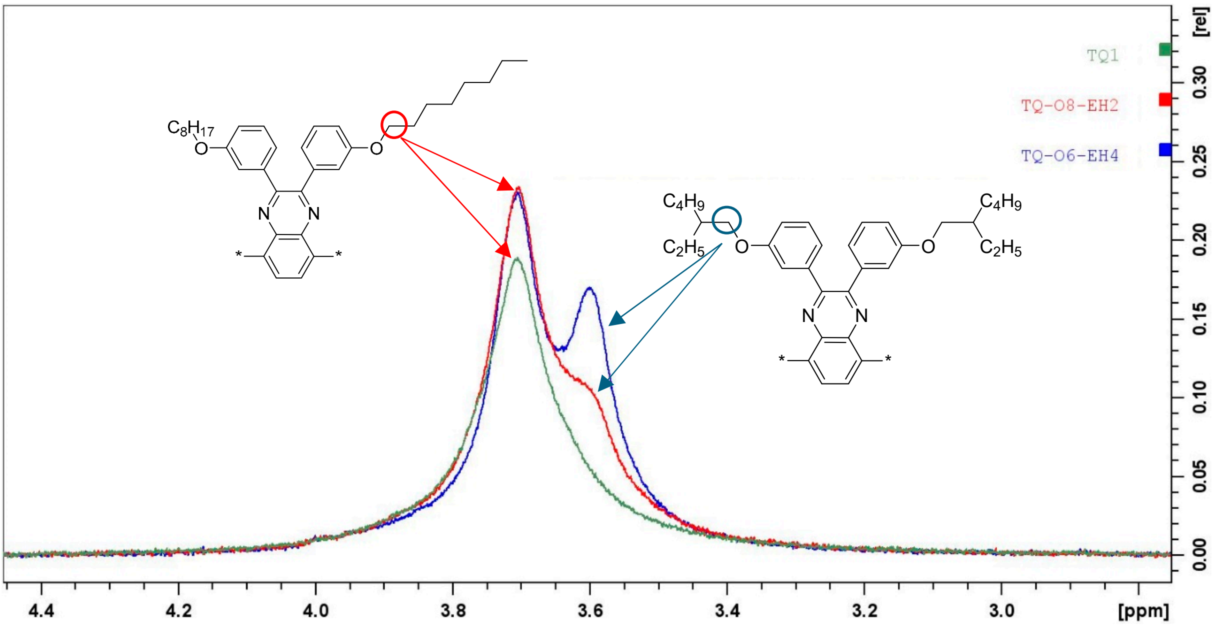


**Figure S2. $^{1}$H NMR (600 MHz) spectra of TQ1 (green curve), TQ-O8-EH2 (red curve) and TQ-O6-EH4 (blue curve). The quinoxaline units with highlighted (-O-$CH_2$-) groups and arrows inserted illustrate the specific proton signals.**

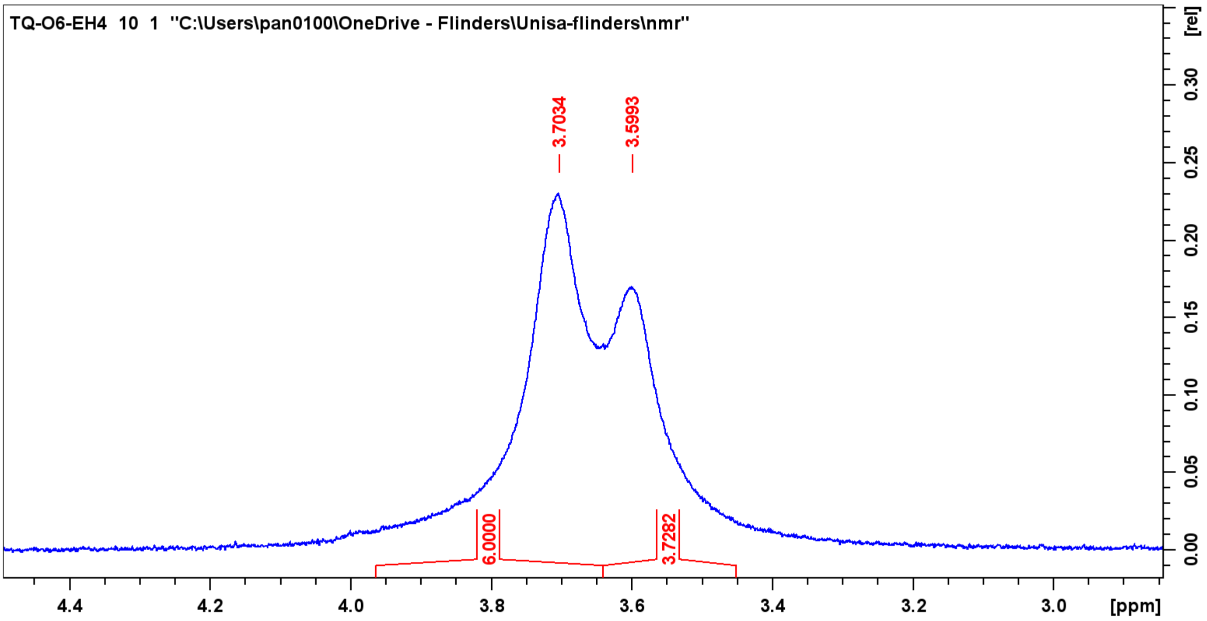


**Figure S3.** **¹H NMR (600 MHz) spectra of TQ-O6-EH4. The integrals of the (-O-$CH_2$-) proton signals reveals that the ratio of Q-O to Q-EH is 6:3.7.**

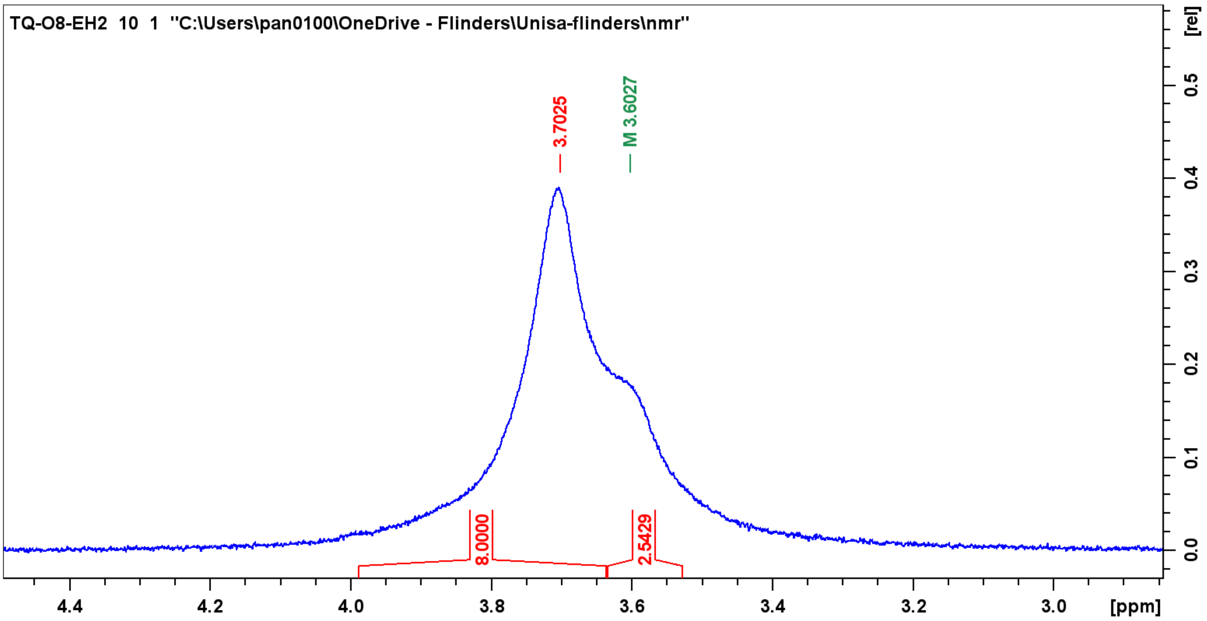


**Figure S4.** **¹H NMR (600 MHz) spectra of TQ-O8-EH2. The integrals of the (-O-$CH_2$-) proton signals reveals that the ratio of Q-O to Q-EH is 8:2.5.**

# 3. GPC traces

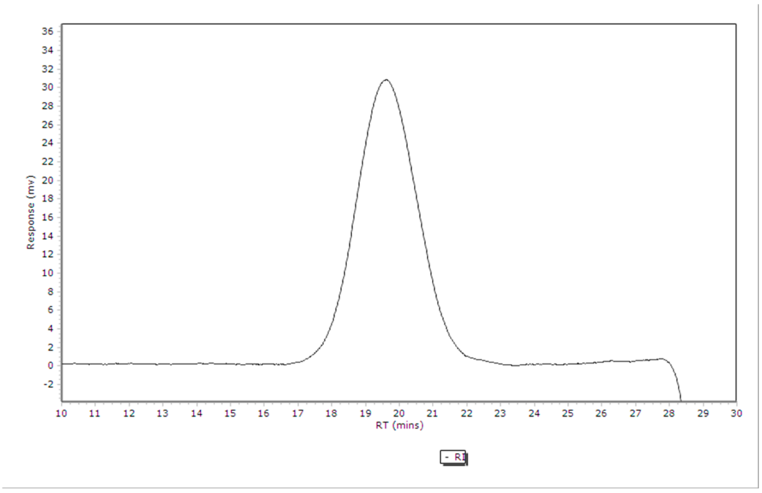


Molecular Weight Averages

| Peak | Mp | Mn | Mw | Mz | Mz+1 | Mv | PD |
|---|---|---|---|---|---|---|---|
| Peak 1 | 47709 | 28396 | 68905 | 151487 | 290868 | 135831 | 2,427 |

**Figure S5. GPC curve of TQ-EH.**

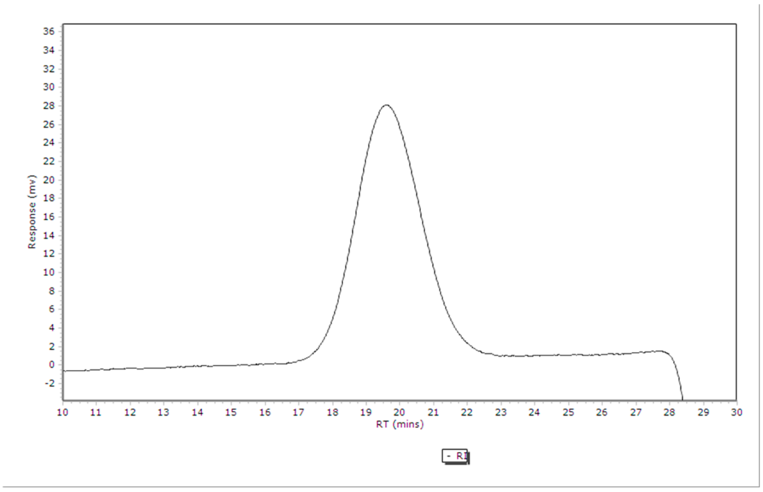


Molecular Weight Averages

| Peak | Mp | Mn | Mw | Mz | Mz+1 | Mv | PD |
|---|---|---|---|---|---|---|---|
| Peak 1 | 47709 | 25358 | 69847 | 170242 | 394002 | 149777 | 2,754 |

**Figure S6. GPC curve of TQ-O2-EH8 *o*-DCB extracts.**

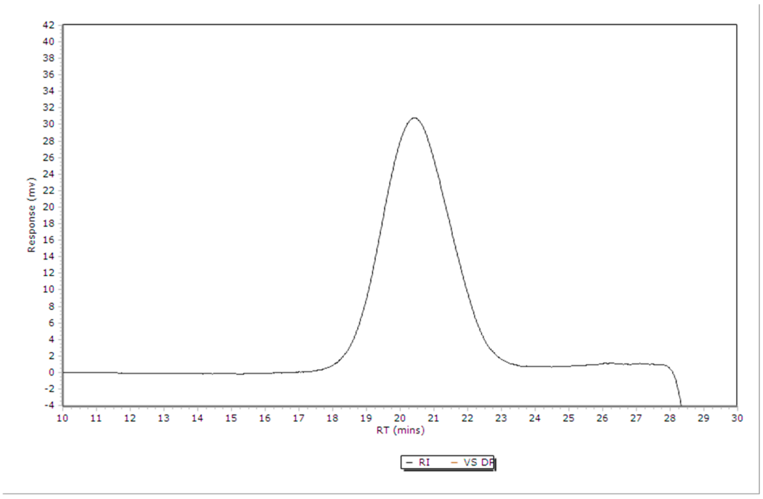


**Molecular Weight Averages**

| Peak | Mp | Mn | Mw | Mz | Mz+1 | Mv | PD |
|---|---|---|---|---|---|---|---|
| Peak 1 | 19563 | 10521 | 29732 | 72806 | 145804 | 64650 | 2,826 |

**Figure S7. GPC curve of TQ-O4-EH6 $CHCl_3$ extracts.**

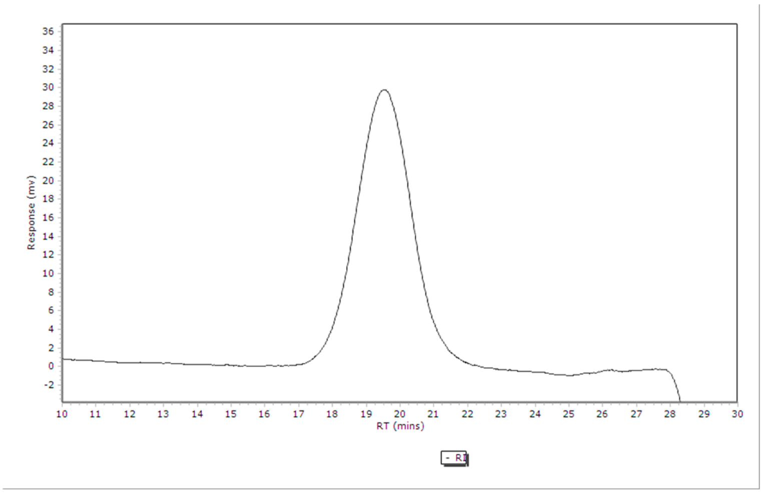


**Molecular Weight Averages**

| Peak | Mp | Mn | Mw | Mz | Mz+1 | Mv | PD |
|---|---|---|---|---|---|---|---|
| Peak 1 | 51310 | 31232 | 74695 | 163589 | 359728 | 145070 | 2,392 |

**Figure S8. GPC curve of TQ-O4-EH6 *o*-DCB extracts.**

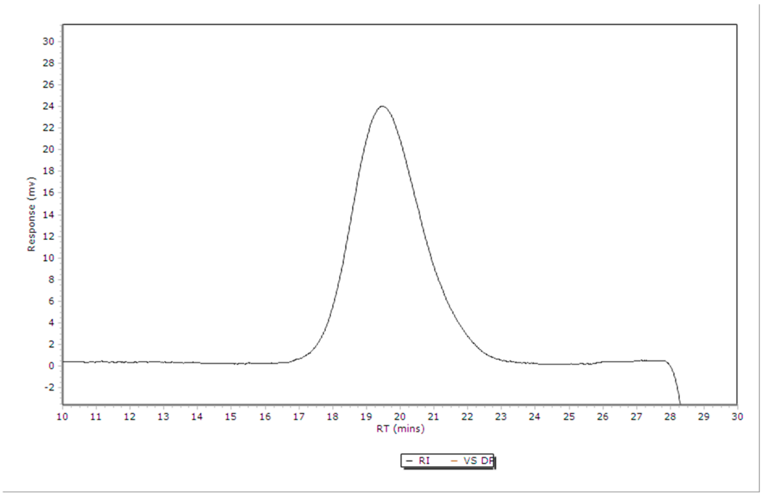


**Molecular Weight Averages**

| Peak | Mp | Mn | Mw | Mz | Mz+1 | Mv | PD |
|---|---|---|---|---|---|---|---|
| Peak 1 | 54189 | 21928 | 75737 | 197234 | 428054 | 173368 | 3,454 |

**Figure S9. GPC curve of TQ-O6-EH4.**

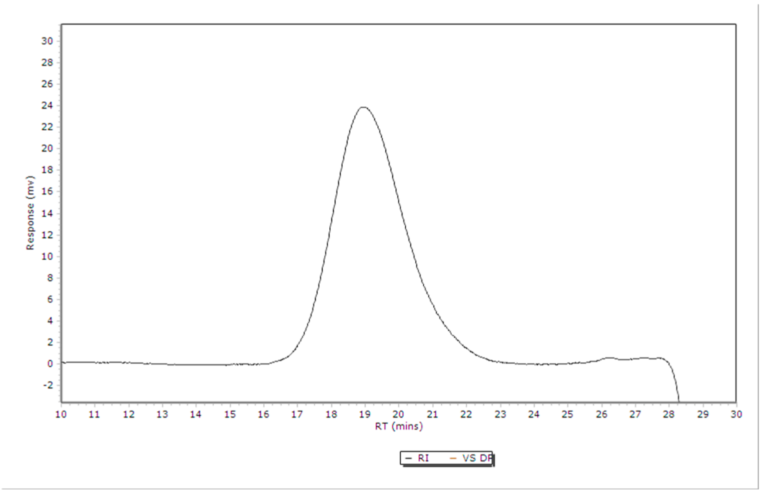


**Molecular Weight Averages**

| Peak | Mp | Mn | Mw | Mz | Mz+1 | Mv | PD |
|---|---|---|---|---|---|---|---|
| Peak 1 | 95246 | 32185 | 131205 | 350085 | 773779 | 307428 | 4,077 |

**Figure S10. GPC curve of TQ-O8-EH2.**

# 4. UV-vis spectra of polymers

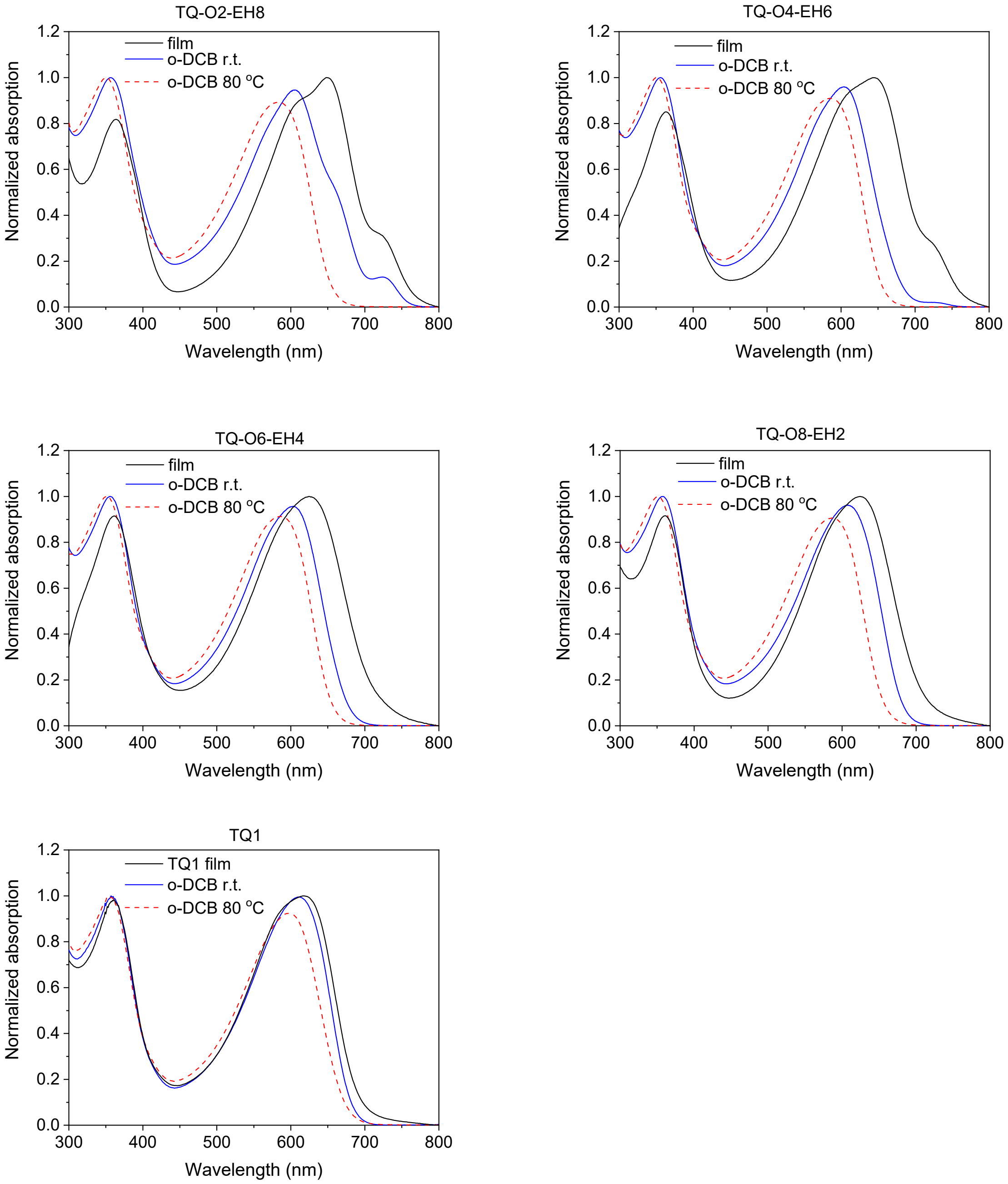


**Figure S11. UV-vis spectra of polymers in different conditions including 80 °C and r.t. *o*-DCB solutions and solid films.**

# 5. Electrochemistry

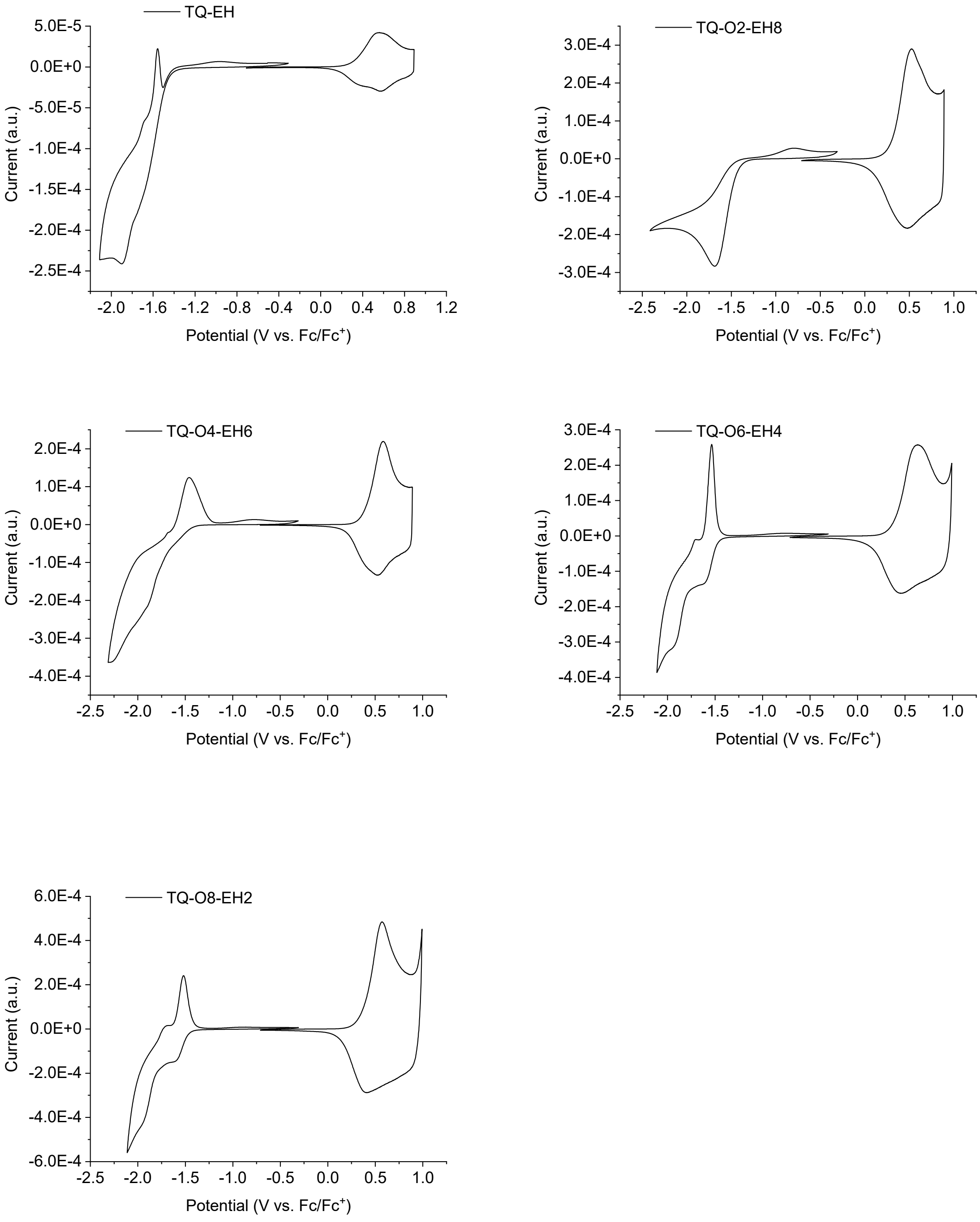


**Figure S12. Cyclic voltammograms of copolymers.**

## 6. DMTA of TQ-O2-EH8 under different frequencies

Gaussian model was used to fit the loss moduli (E”) of TQ-O2-EH8 measured under the frequencies of 0.1, 1, 6.3 and 10 Hz.

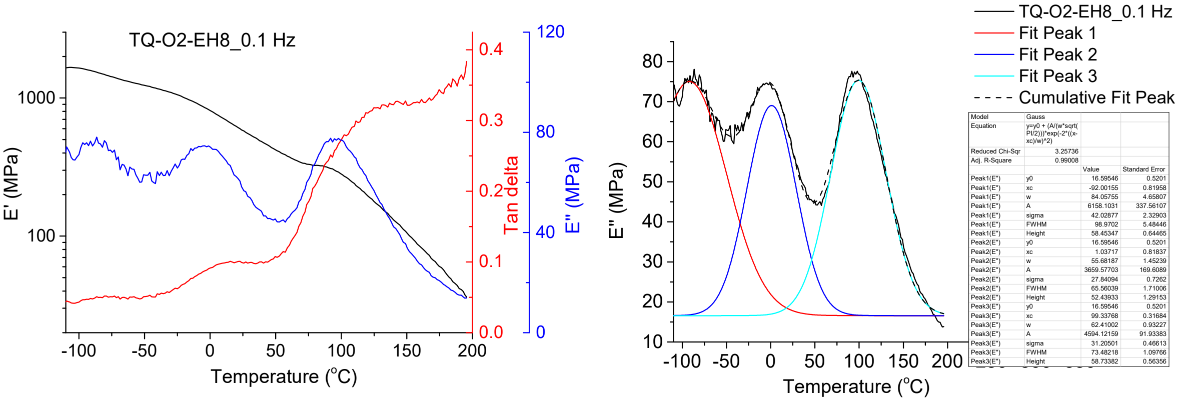


**Figure S13. DMTA plot TQ-O2-EH8 measured at 0.1 Hz. Peak fitting of E” of TQ-O2-EH8 at 0.1 Hz.**

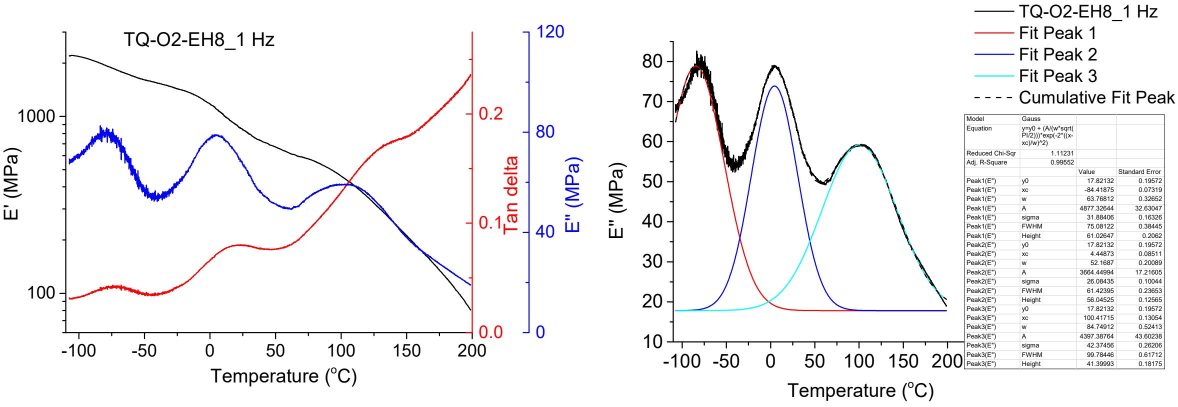


**Figure S14. DMTA plot TQ-O2-EH8 measured at 1 Hz. Peak fitting of E” of TQ-O2-EH8 at 1 Hz.**

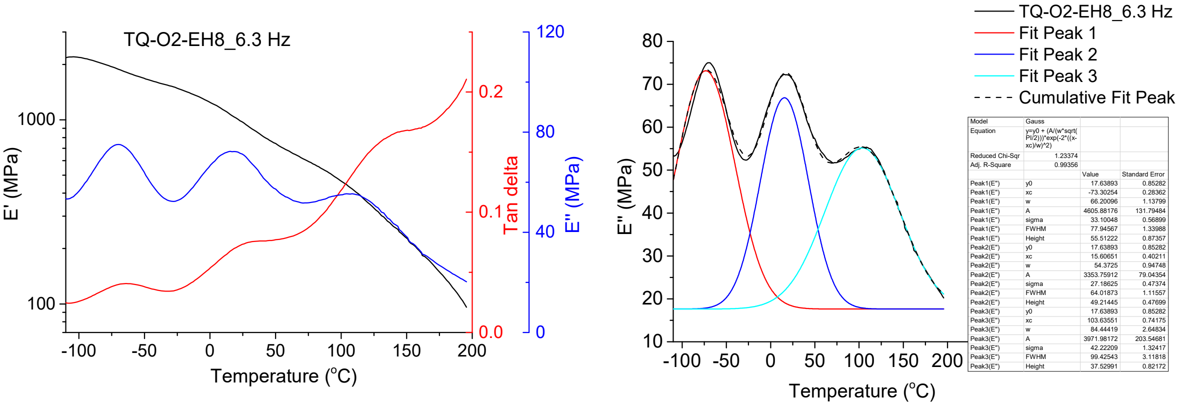


**Figure S15. DMTA plot TQ-O2-EH8 measured at 6.3 Hz. Peak fitting of E” of TQ-O2-EH8 at 6.3 Hz.**

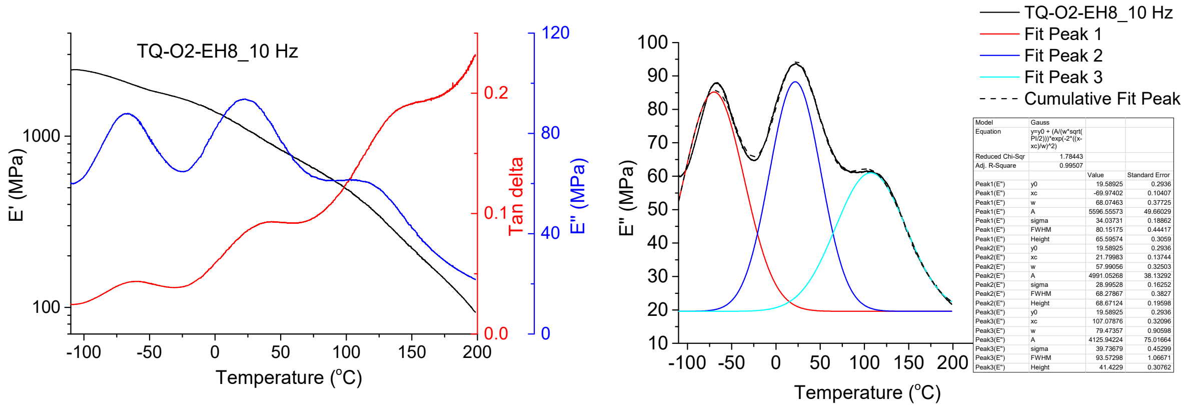


**Figure S16. DMTA plot TQ-O2-EH8 measured at 10 Hz. Peak fitting of E” of TQ-O2-EH8 at 10 Hz.**

# 7. DSC thermograms

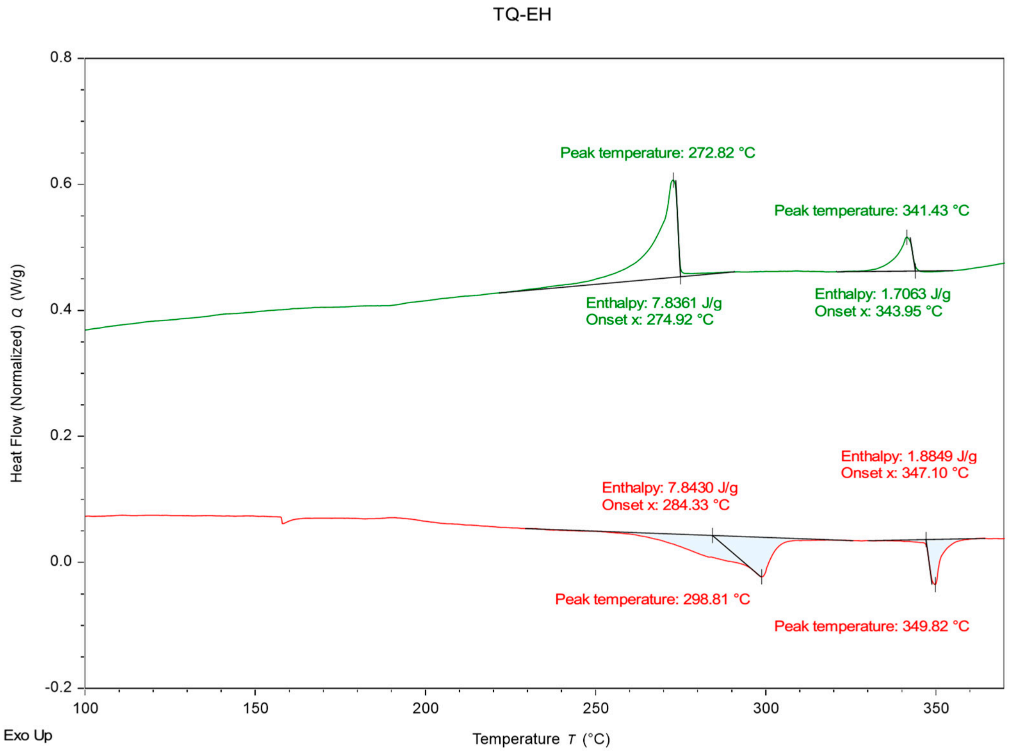


**Figure S17. DSC 2nd heating (red curve) and cooling (green curve) thermograms of TQ-EH.**

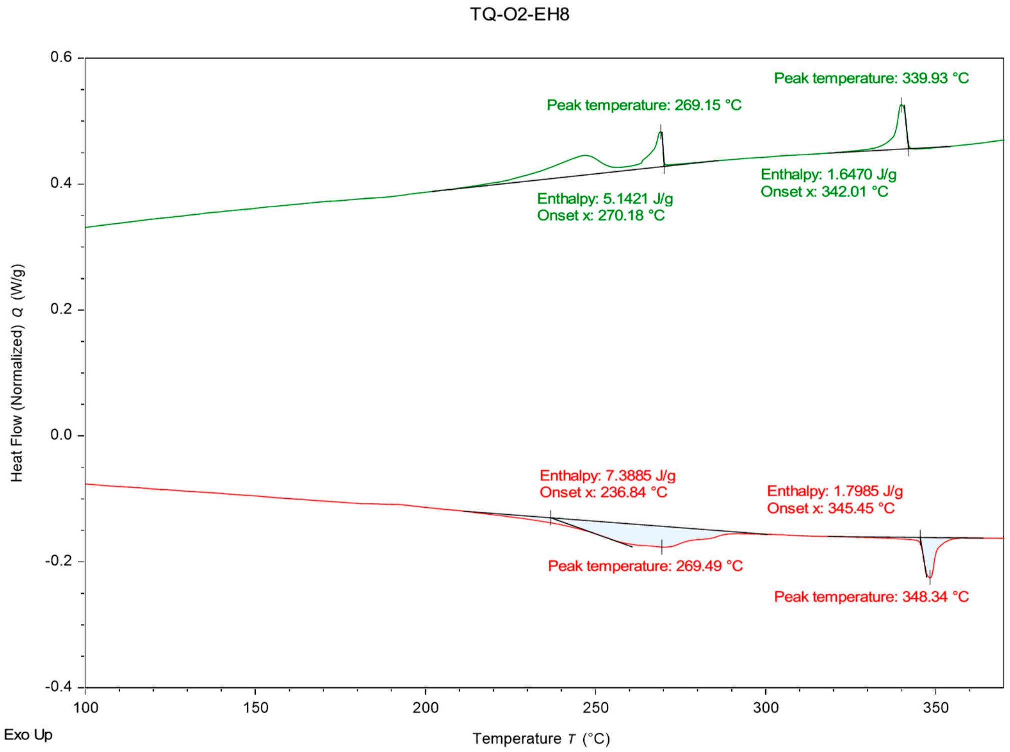


**Figure S18. DSC 2nd heating (red curve) and cooling (green curve) thermograms of TQ-O2-EH8.**

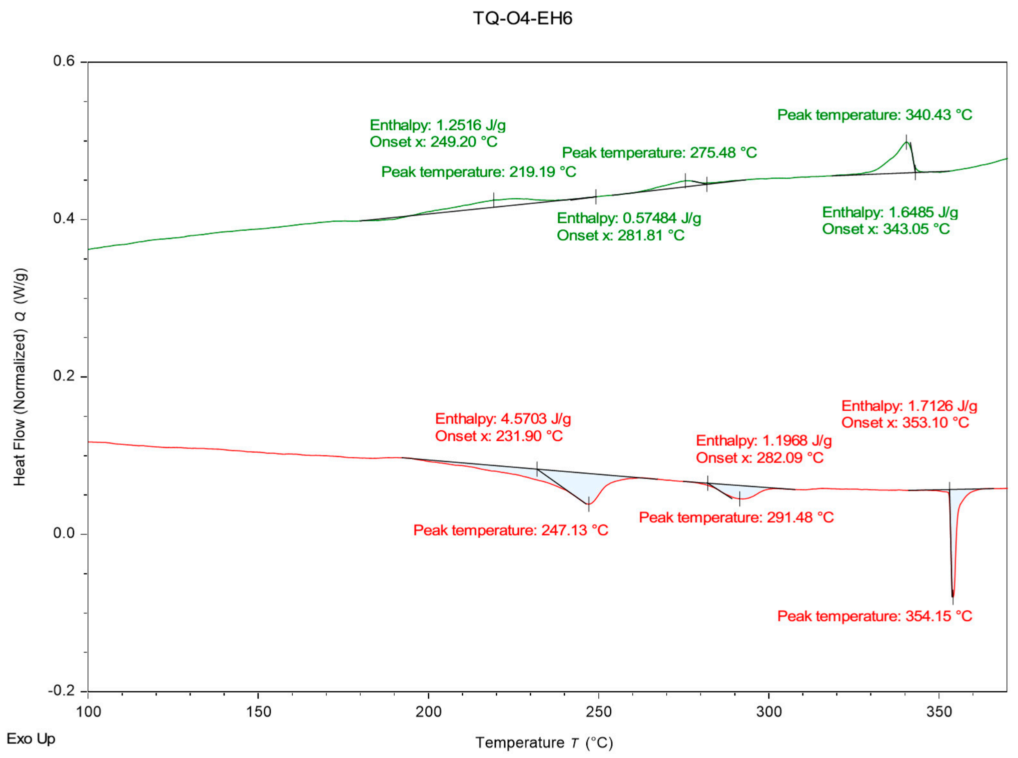


**Figure S19. DSC 2nd heating (red curve) and cooling (green curve) thermograms of TQ-O4-EH6.**

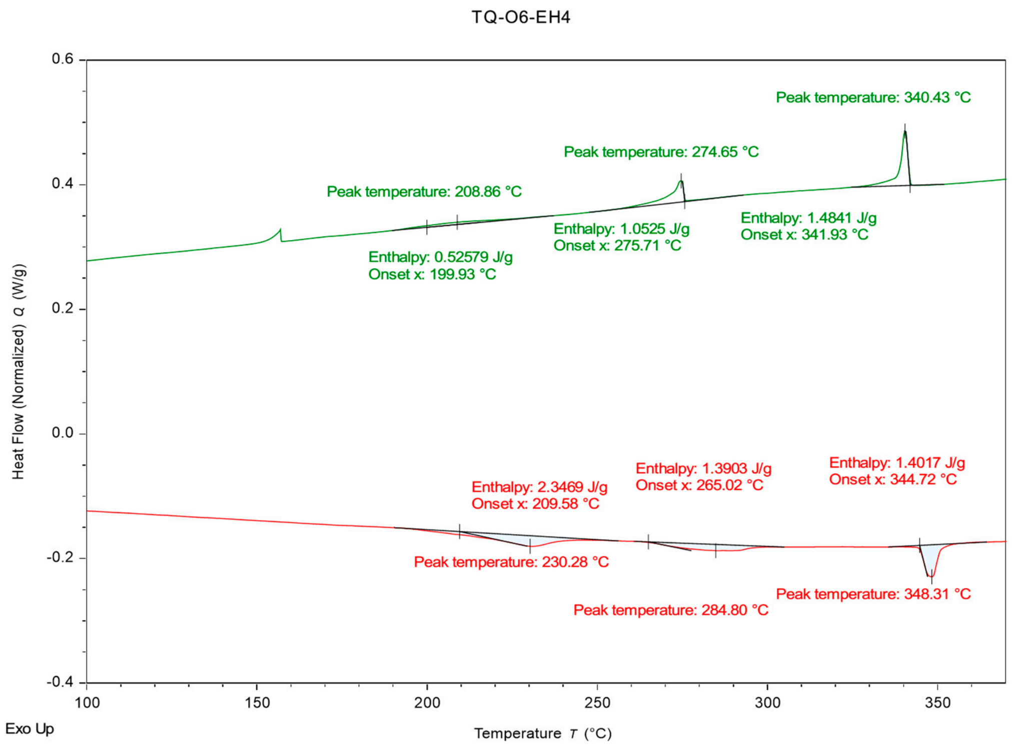


**Figure S20. DSC 2nd heating (red curve) and cooling (green curve) thermograms of TQ-O6-EH4.**

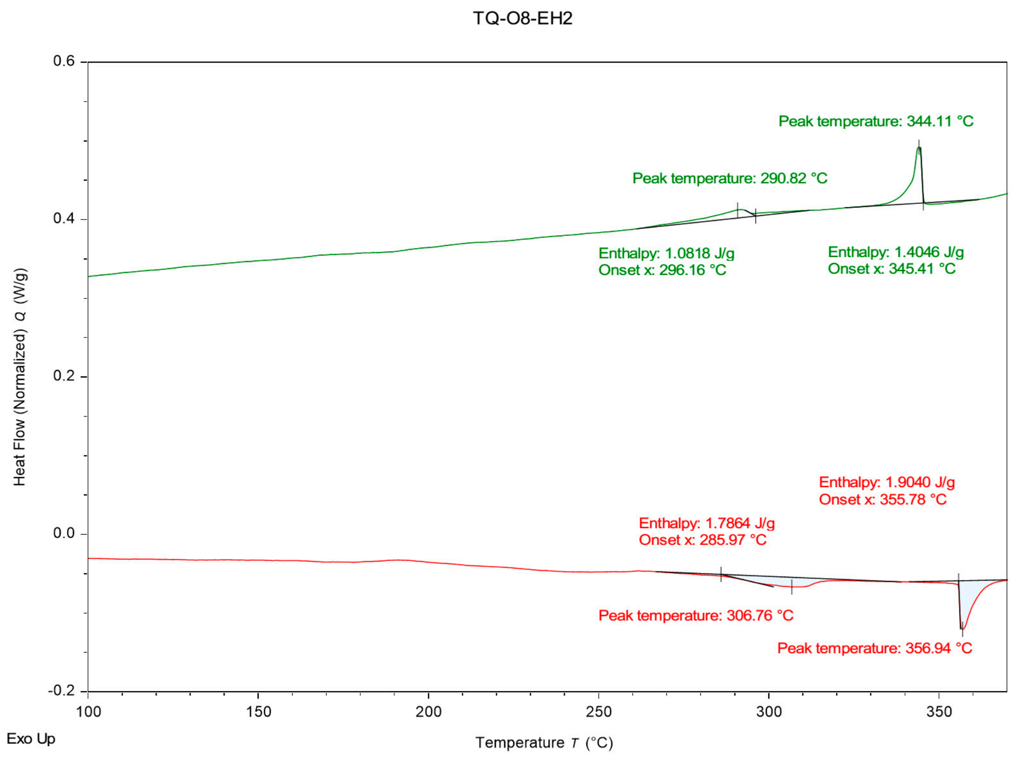


**Figure S21. DSC 2nd heating (red curve) and cooling (green curve) thermograms of TQ-O8-EH2.**

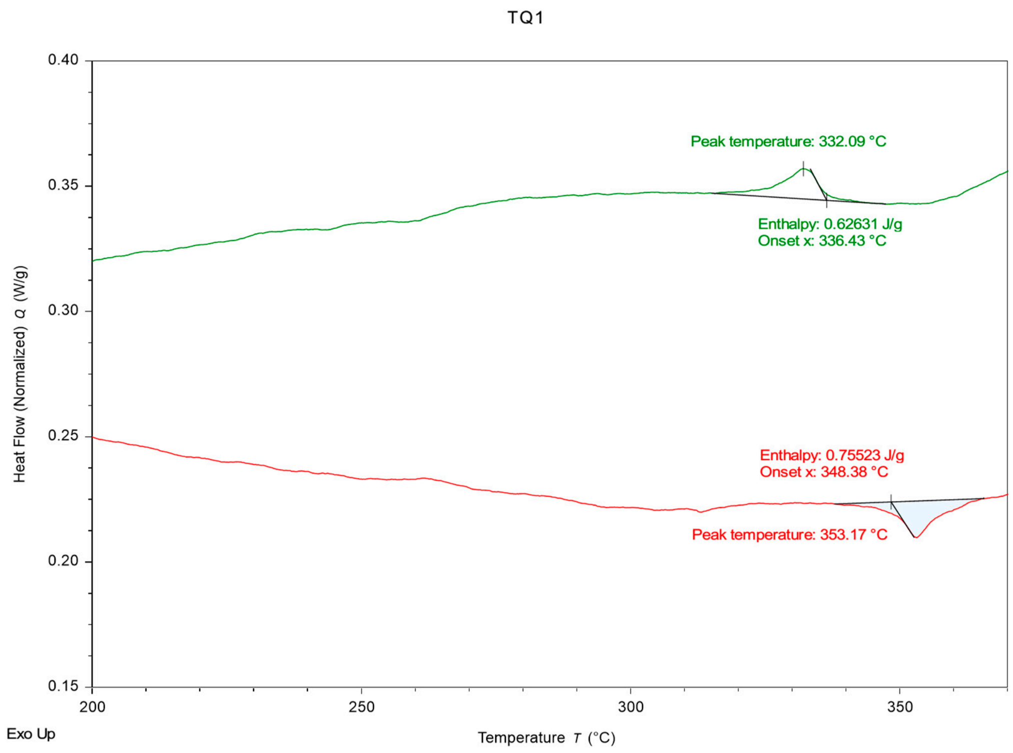


**Figure S22. DSC 2nd heating (red curve) and cooling (green curve) thermograms of TQ1.**

**Table S2. Melting transition peak temperature ($T_m$), the liquid-crystalline to isotropic transition temperature ($T_{LC}$) and the corresponding enthalpy ($\Delta H_{LC}$) obtained from the DSC second heating thermograms.**

| Polymer | EH side-chain content | $T_m$ (°C) | $T_{LC}$ (°C) | $\Delta H_{LC}$ (J/g) |
|---|---|---|---|---|
| TQ-EH | 100% | 298.1 | 349.8 | 1.88 |
| TQ-O2-EH8 | 80% | 269.5 | 348.3 | 1.80 |
| TQ-O4-EH6 | 60% | 247.1, 291.5 | 354.2 | 1.71 |
| TQ-O6-EH4 | 40% | 230.3, 284.8 | 348.3 | 1.40 |
| TQ-O8-EH2 | 20% | 306.8 | 356.9 | 1.90 |
| TQ1 | 0% | - | 353.2 | 0.76 |

# 8. EQE of TQ-O4-EH6:$PC_{71}BM$ device

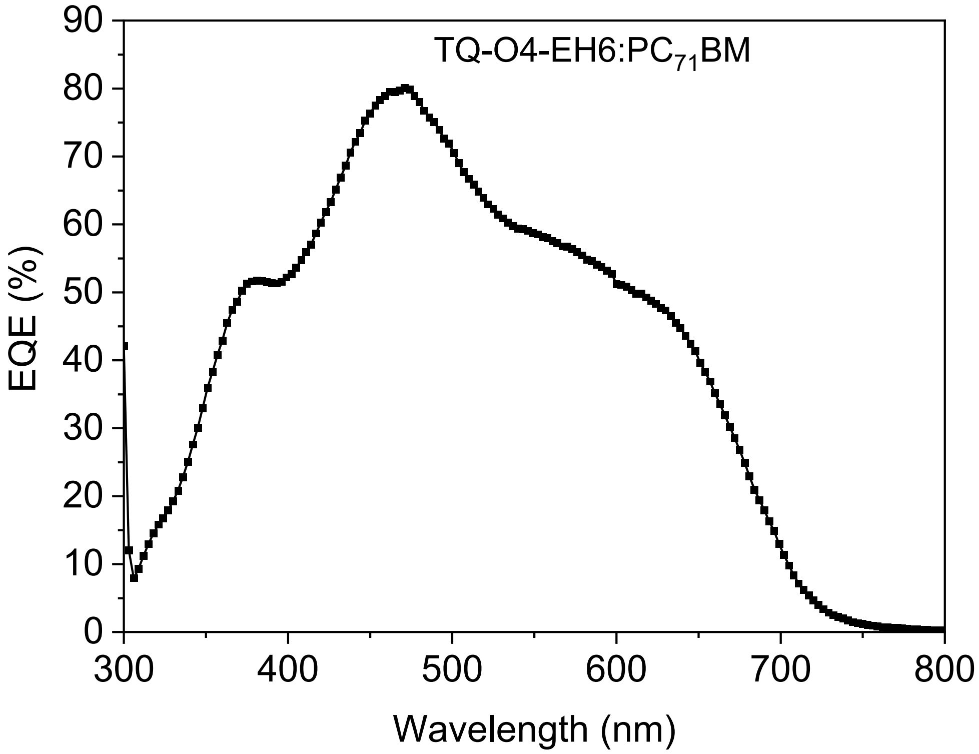


**Figure S23. EQE curve of TQ-O4-EH6:$PC_{71}BM$ device.**

# 9. AFM height and phase images

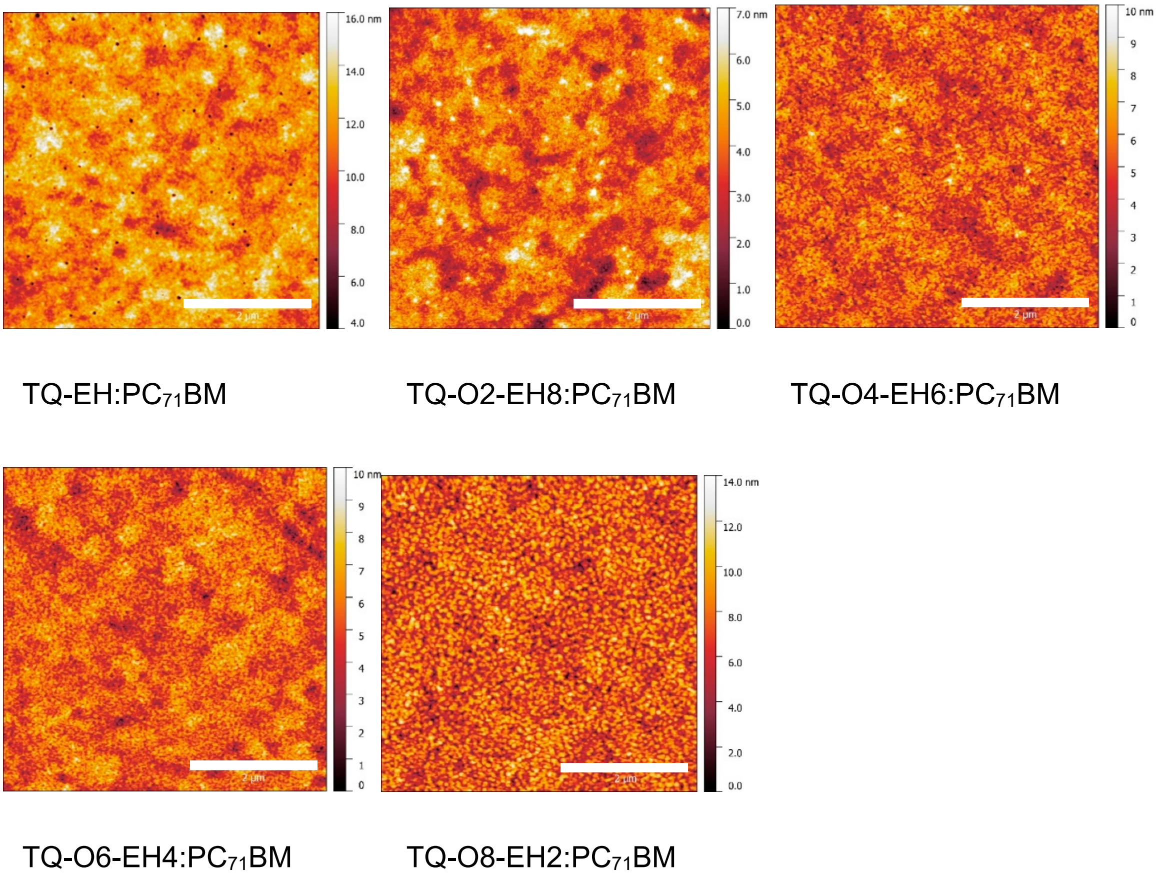


**Figure S24. 5 × 5 µm$^2$ AFM height images of polymer:PC$_{71}$BM. Scale bars are 2 µm.**

**Table S3. root-mean square roughness (RMS) values calculated from the 5 × 5 µm$^2$ polymer:PC$_{71}$BM height images.**

| **Polymer** | **RMS(nm) (5 x 5 µm)** |
|---|---|
| TQ-EH | 1.24 |
| TQ-O2-EH8 | 0.90 |
| TQ-O4-EH6 | 1.13 |
| TQ-O6-EH4 | 1.12 |
| TQ-O8-EH2 | 1.94 |

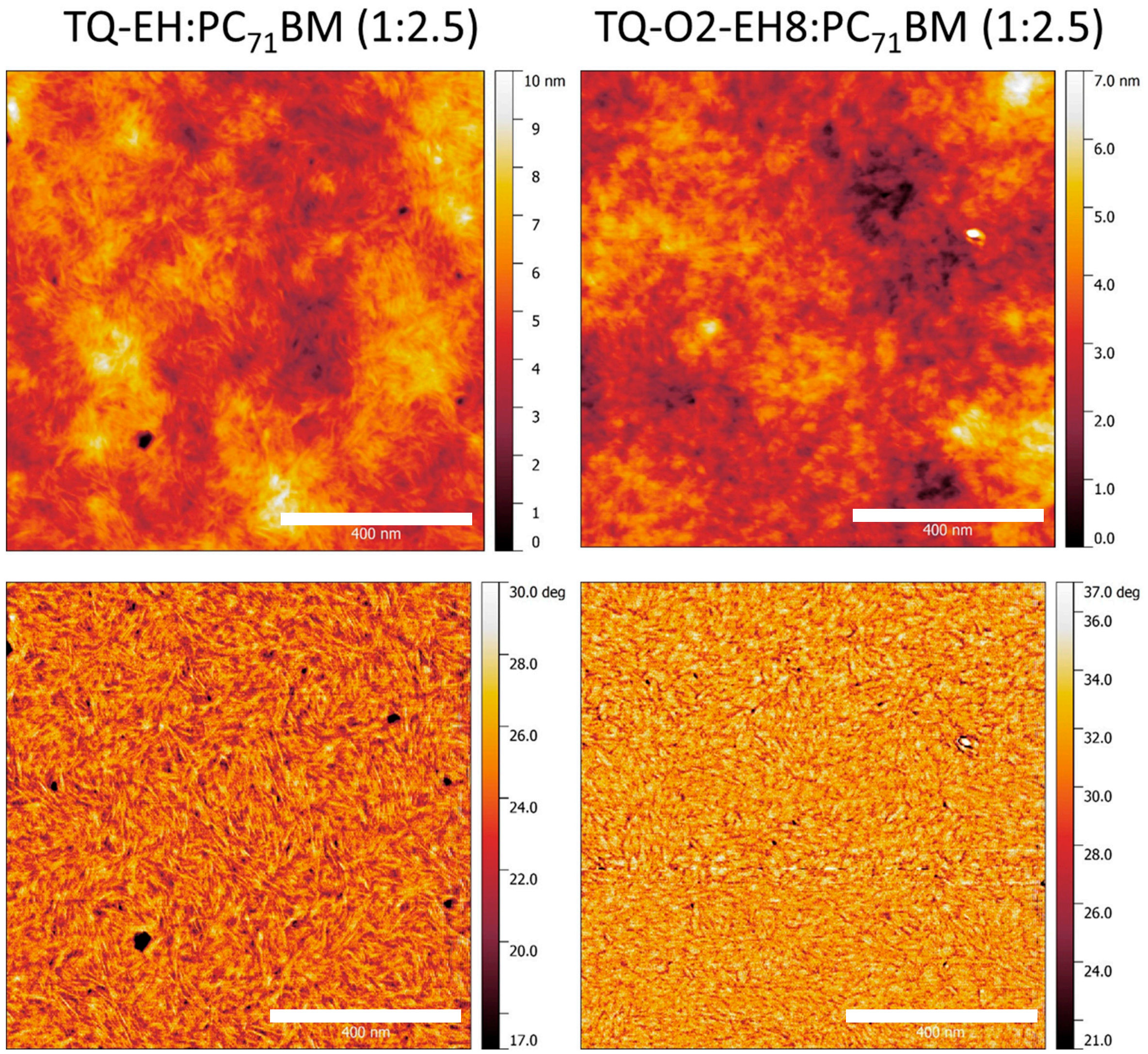


**Figure S25.** 1 × 1 μm[2] AFM height and phase images of TQ-EH:$PC_{71}BM$ and TQ-O2-EH8:$PC_{71}BM$ films. Scale bars are 400 nm.

# 10. References

(1) Pan, X.; Sharma, A.; Kroon, R.; Gedefaw, D.; Elmas, S.; Yin, Y.; Andersson, G. G.; Lewis, D. A.; Andersson, M. R. Water/Ethanol Soluble p-Type Conjugated Polymers for the Use in Organic Photovoltaics. *Frontiers in Materials* **2020**, *7*. DOI: 10.3389/fmats.2020.00281.
